# *Clust*: automatic extraction of optimal co-expressed gene clusters from gene expression data

**DOI:** 10.1186/s13059-018-1536-8

**Published:** 2018-10-25

**Authors:** Basel Abu-Jamous, Steven Kelly

**Affiliations:** Department of Plant Sciences, University of Oxford, South Parks Road, Oxford, OX1 3RB UK

**Keywords:** Clustering, Gene expression data, *Clust*, K-means, Cross-clustering, Click, Markov clustering, Hierarchical clustering, Self-organizing maps, WGCNA

## Abstract

Identifying co-expressed gene clusters can provide evidence for genetic or physical interactions. Thus, co-expression clustering is a routine step in large-scale analyses of gene expression data. We show that commonly used clustering methods produce results that substantially disagree and that do not match the biological expectations of co-expressed gene clusters. We present *clust*, a method that solves these problems by extracting clusters matching the biological expectations of co-expressed genes and outperforms widely used methods. Additionally, *clust* can simultaneously cluster multiple datasets, enabling users to leverage the large quantity of public expression data for novel comparative analysis. *Clust* is available at https://github.com/BaselAbujamous/clust.

**Electronic supplementary material:**

The online version of this article (10.1186/s13059-018-1536-8) contains supplementary material, which is available to authorized users.

## Background

Gene transcription is dynamically and coordinately regulated in all living organisms. Such coordinate regulation is manifest as concordant changes in the transcript abundance of genes in time series and perturbation-response datasets. Gene transcription is regulated by the binding of transcription factors to DNA/chromatin elements located in promoter or enhancer regions of genes. Typically, transcription factors comprise ~ 10% of the total number of genes in a genome, and complex spatio-temporal patterns of transcription are achieved through the combinatorial action of these genes in regulatory networks [1]. The combinatorial nature of these networks means that their behavior is inherently conditional. That is, genes that appear co-expressed under one condition are not necessarily co-expressed under all conditions. A corollary of this is that within any one experimental context (e.g., time series spanning some biological process or perturbation-response experiment), not all genes will be behaving coordinately. Instead, subsets of genes will have the right combination of regulators to behave coordinately during the experimental context while others are following patterns of regulation that are independent of the experimental design. Thus, within a given observation window (i.e., experimental context), it is not expected that all genes can be assigned to a limited set of coordinate behavior [2, 3].

Given that only subsets of genes are likely to be co-expressed within a particular context, it follows that identification of these subsets is a data extraction problem and not a data partitioning problem. That is, the aim of gene expression clustering is to identify and extract the cohorts of genes that are behaving coordinately from the complete set of genes that are detected within a particular context, and is not to partition the complete set of genes into a set of gene clusters. In practice, clustering methods have been widely applied to gene expression data with the expectation that they will identify the complete set of discrete cohorts of genes that have co-ordinated behavior (i.e., the clusters of co-expressed genes), and that all of genes that exhibit those behavior will be assigned to the correct cluster [4, 5]. However, the vast majority of methods that aim to identify cohorts of co-expressed genes are based on data partitioning (e.g., k-means [6], hierarchical clustering [7], and self-organizing maps [8]). These approaches attempt to assign all genes to a finite set of clusters, with the number of clusters determined by numerical optimization of a data partitioning metric [9]. Thus, genes that are not co-expressed in the context under investigation are also assigned to their “best-fitting” cluster such that the majority of clusters will contain both co-expressed and non-co-expressed genes. This result does not adhere to the expectation of the biological properties of a co-expressed gene cluster, i.e., that each cluster contains only those genes that exhibit co-ordinate behavior in the experimental or biological context under question and that no two clusters should have an identical profile. Although data partitioning methods are most commonly used, a number of partial clustering methods have also been proposed [5, 10–12]. These methods do not require that the complete data set is partitioned between clusters; instead, they aim to identify the subset that can be readily assigned to clusters.

Here we show through analysis of 100 real biological datasets from five model organisms that application of data partitioning-based and partial clustering-based methods to gene expression data generates clusters that include substantial numbers of unreliably assigned genes, i.e., genes that do not exclusively fit in their clusters and should have been excluded. Such unreliable content comprises up to about 50% of these clusters. To address this problem, we provide a novel method called *clust* for cluster extraction from gene expression data. *Clust* is designed to extract co-expressed clusters of genes that satisfy the biological expectations of a co-expressed gene cluster. We show that *clust* satisfies these expectations by extracting co-expressed clusters with lower levels of dispersion than data partitioning methods and partial clustering methods. We also show that the clusters produced by *clust* are better than those produced by any other tested method by 7 different measures of clustering performance. Furthermore, we show that the clusters extracted by *clust* are equally, or more, significantly enriched with functional terms than those produced by other methods. Finally, we demonstrate the ability of *clust* to extract clusters of consistently co-expressed genes in multiple datasets simultaneously, enabling researchers to leverage multiple disparate datasets to identify high accuracy co-expressed gene clusters.

## Results

### Problem definition, aim, and approach

Gene expression datasets (RNA-seq and microarray) contain quantitative estimates (observations) of mRNA abundance for a set of genes at multiple experimentally, spatially, or temporally discrete conditions. Across these conditions, it is expected that the mRNA abundance of transcriptionally co-regulated genes will exhibit coordinate behavior. These co-regulated cohorts of genes include those that are inherent modules of the system being studied, as well as those that may be conditional on applied experimental perturbations. The observations also include transcript abundance estimates for genes that are behaving independently in the experimental series. Furthermore, for genes that are transcriptionally co-regulated, variance in RNA processing and mRNA half-life cause fluctuations in transcript abundance such that abundance estimates are inherently noisy. Thus, the goal of gene expression clustering is to identify and extract the discrete cohorts of genes whose transcripts are behaving coordinately (albeit with biological noise) across the observations under consideration.

**Fig. 1:**
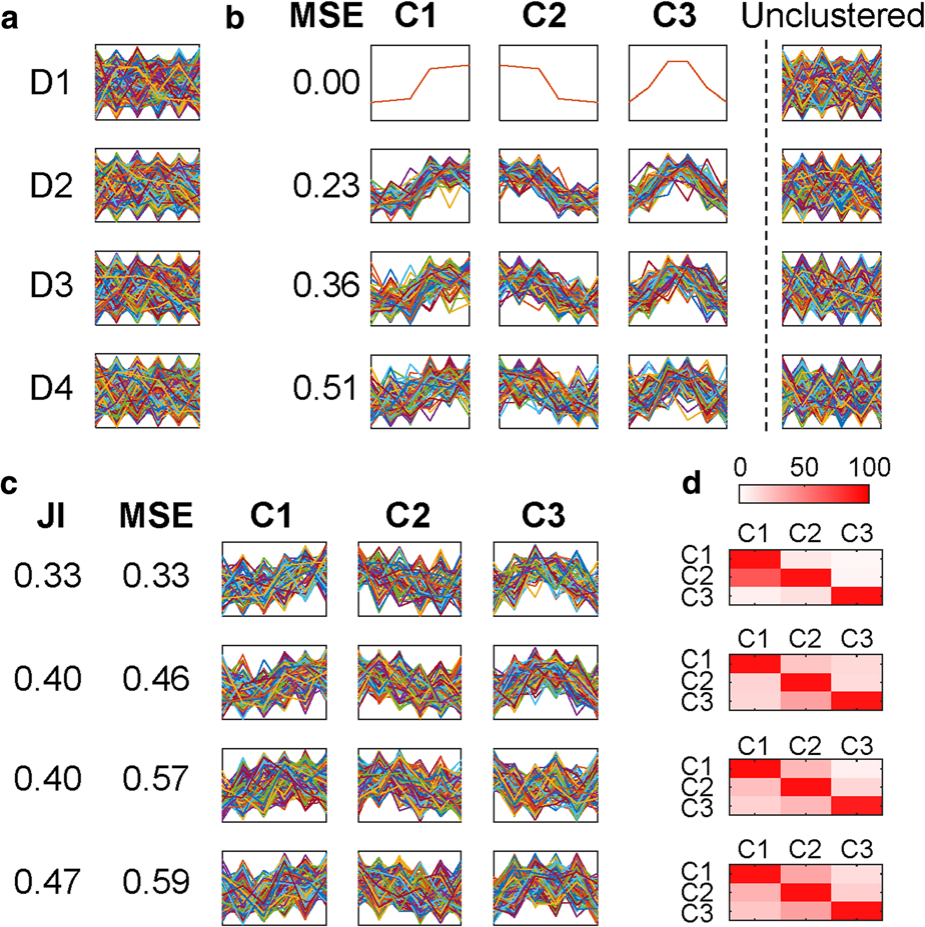
Expectations and outcomes for application of data-partitioning methods to co-expression clustering. **a**, **b** Simulated gene expression data for 500 genes with increasing noise (D1–D4) (Additional file 1: Table S1). **a** All genes. **b** Profiles of the genes in each of the three simulated clusters as well as the extra unclustered genes at each one of the four levels of dispersion. The horizontal axis of each plot represents the six different time-points, while the vertical axis represents gene expression values. **c** The results of applying a partitioning method (*k*-means in this case) to the same simulated datasets. **d** Heat-maps that show the percentage of genes in a cluster that also fit well within each one of the other clusters

Figure 1 presents simulated gene expression data to illustrate the problem of extracting distinct cohorts of co-expressed genes. Each simulated dataset contains 500 genes, with 100 genes in each of three distinct clusters and 200 genes that do not belong to any cluster. Detailed description of these datasets is provided in the “Methods” section, and their values are provided in Additional file 1: Table S1. Figure 1a shows the same datasets simulated with increasing levels of biological noise (D1 to D4), and Fig. 1b shows the desired results. That is, to extract three distinct clusters of genes (C1 to C3) while discarding the genes that behave independently. In conflict with the desired goal, data partitioning methods require all genes to be included in one of the clusters. For example, application of *k*-means (the most commonly used method for analyzing gene expression datasets) recovers the three simulated profiles (Fig. 1c). However, each cluster also contains a large cohort of genes that do not share the same expression profile (Fig. 1d). This inclusion results in clusters with high levels of dispersion (differences in expression levels between genes within a cluster) and high levels of inter-cluster similarity, violating the expectations of co-expressed gene clusters, and producing clusters whose gene assignment is unreliable. *Clust* is designed to address this problem by extracting the largest and least dispersed set of clusters whose profiles are distinct and exclude those genes that do not belong to these clusters. That is, to identify and extract the complete set of genes that are exhibiting coordinate behavior in the experimental series under consideration. The results of applying *clust* to these demonstrative datasets are included in Additional file 2: Figure S1.

### The clust method

**Fig. 2:**
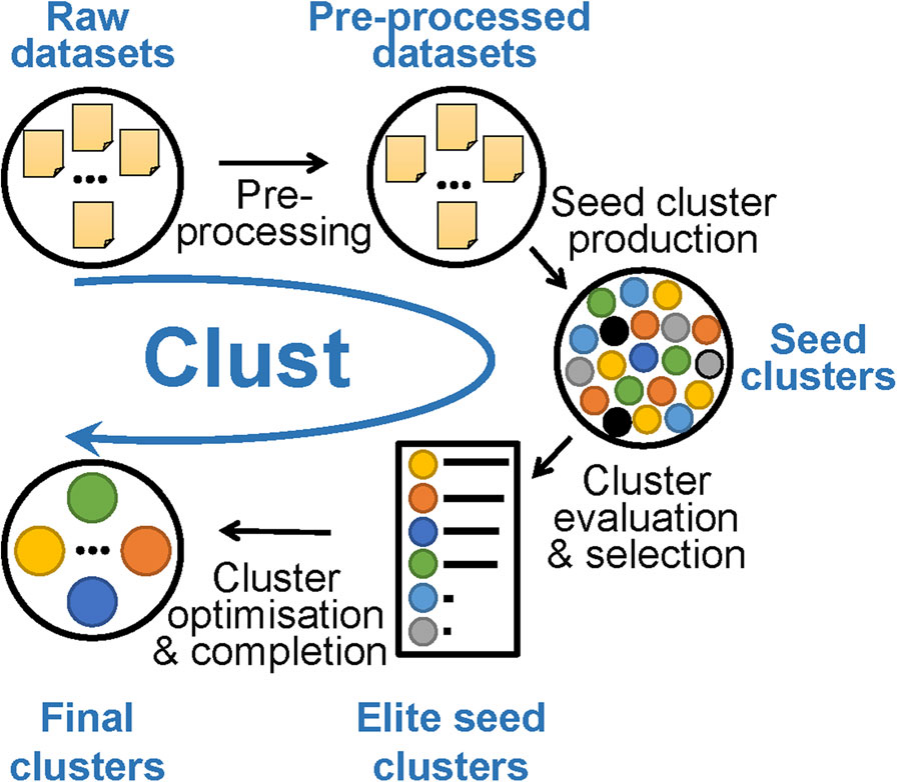
Pipeline of the steps of the *clust* method. The *clust* pipeline is composed of four major steps: (1) data pre-processing of the one or more input raw datasets, (2) production of a pool of seed clusters, (3) cluster evaluation and the selection of a subset of elite seed clusters, and (4) the optimization and completion of the elite seed clusters to produce final clusters

Figure 2 shows an overview pipeline of the steps composing the *clust* method. The method takes one or more datasets as an input. The first step is pre-processing the datasets by summarizing replicates, filtering out genes with low expression, and normalizing gene expression values as required. The user may choose their preferred pre-processing options, but the best practice options are indicated in the description of the publically available *clust* python package online. After that, *clust* produces a pool of “seed clusters” by applying *k*-means clustering multiple times to this data with different *K* values. If the input includes more than one dataset, consensus clusters over these datasets are calculated using the binarization of consensus partition matrices (Bi-CoPaM) method [13]. These seed clusters are then evaluated by the M-N scatter plots technique [14], and elite seed clusters are selected. This technique favours clusters of larger sizes that maintain low dispersion values and guarantees that clusters are distinct. Finally, the elite seed clusters are analyzed to learn the distributions of within-cluster dispersion in the selected datasets; this information is used to remove outliers from clusters and identify genes that fit within clusters but that have been missed by the previous steps. A full description of the *clust* algorithm is provided in Additional file 3: Text S1. A standalone Python implementation of *clust* is available at https://github.com/BaselAbujamous/clust [15].

### Data sources and comparative methods

To demonstrate the performance characteristics of *clust* on real biological datasets, the method was applied to 100 different gene expression datasets (Additional file 4: Table S2). These datasets comprised ten microarray datasets and ten RNA-seq datasets from each of five different model organisms: *Homo sapiens*, *Mus musculus*, *Drosophila melanogaster*, *Arabidopsis thaliana*, and *Saccharomyces cerevisiae*. To put these performance characteristics in context, seven of the most commonly used co-expression clustering methods (Cross Clustering (CC) [12], *k*-means [6], self-organizing maps (SOMs) [8], Markov clustering (MCL) [16], hierarchical clustering (HC) [7], Click [10], and WGCNA [17]) were also applied to these datasets. For each of these comparative methods, the best-practice operating procedures were followed as described in the “Methods” section. In all cases, the data pre-processing procedures were the same for each method and were applied as described in the “Methods” section.

### Clust robustly extracts tight and non-overlapping clusters

**Fig. 3:**
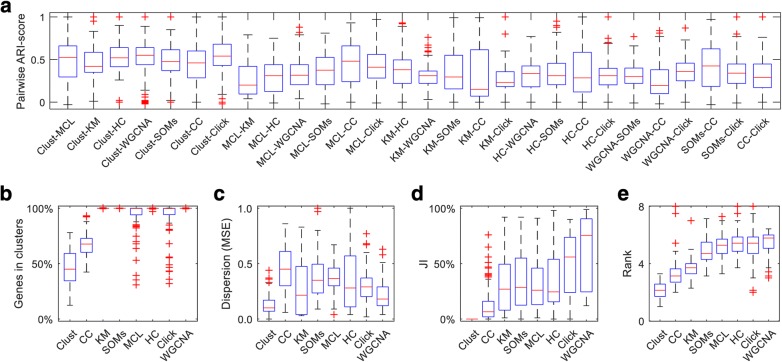
Evaluation of the performance of clustering methods. **a** Similarity of clustering results generated by each pair of methods measured by the Adjusted Rand Index (ARI); 1.0 means exactly similar and 0.0 means completely dissimilar. **b**–**d** Evaluation of clustering performance over all 100 datasets. **b** The percentage of input genes that were included in clusters; **c** the average dispersion of clusters measured by weighted-averaging of individual cluster MSE values; **d** percentage of the overlap amongst clusters, as measured by the JI index. **e** Evaluation of clustering performance over all 100 datasets as measured by average rank across 7 cluster validation indices that *clust* does not directly optimize; the indices are Davies–Bouldin (DB) index, Bayesian information criterion (BIC), Silhouette, Calinski-Harabasz (CH) index, Ball and Hall (BH) index, Xu index, and within-between (WB) index (Additional file 2: Figures S4 to S11, Additional files 5, 6, and 9: Tables S3, S4 and S7)

Different clustering methods produce very different results when applied to the same dataset (Fig. 3a; Additional file 5: Table S3). For instance, when any two methods are applied to the same dataset, the results will, on average, only be 37% identical (i.e., adjusted rand index similarity score [18] of 0.37). Therefore, clustering results strongly depend on the method that was applied, which raises the question as to which method performs the best.

As *clust* is a cluster extraction method, it does not necessarily assign all genes to clusters. On average across the 100 test datasets *clust* assigned 50% of the input genes to clusters (Fig. 3b). The CC, MCL, and Click methods also extract clusters without forcing all input genes to be in clusters (Fig. 3b). Importantly, *clust* produced sets of clusters that have significantly lower dispersion than those produced by CC (*p* value 1.5 × 10^−39^), *k*-means (*p* value 3.2 × 10^−10^), SOMs (*p* value 4.8 × 10^−27^), MCL (*p* value 8.4 × 10^−35^), HC (*p* value 8.0 × 10^−16^), Click (*p* value 2.5 × 10^−26^), or WGCNA (*p* value 3.9 × 10^−19^) (Fig. 3c; *p* values obtained from paired *T* test, Additional file 6: Table S4). Clusters produced by *clust* are discrete, such that genes assigned to one cluster do not fit within the profile boundaries of any other cluster (JI = 0 for all clusters, Fig. 3d; see the “Methods” section for the definition of cluster boundaries). This is not the case for the other methods, where 10% to 50% of the genes that are included in a given cluster also fit within the boundaries of at least one other cluster (Fig. 3d). Thus, application of these methods to gene expression data produces clusters that are not discrete and contain between 10% and 50% unreliably assigned genes (Additional file 6: Table S4). Analogous results were obtained when the comparative methods were re-run while optimising the MSE and JI metrics, where applicable (Additional file 2: Figure S2). Notably, datasets with more than 50 samples were excluded from this analysis for logistical reasons. However, a total of 19 datasets would have been included had the number of samples not been limited (Additional file 7: Table S5). Replicating the clustering method comparison over these datasets also shows analogous results to those presented in Fig. 3 (Additional file 2: Figure S3). Thus the improved performance of *clust* relative to other clustering methods is independent of dataset size or the criteria used for competitor dataset optimization.

The properties of *clust*’s clusters are independent of their size, that is, the number of genes contained in a given cluster. In contrast, the properties of clusters returned by the majority of the other methods display a significant dependency on cluster size (Additional file 2: Figures S4 and S5; Additional file 8: Table S6). None of the eight methods, including *clust*, behaves differently on datasets from different species (Additional file 2: Figures S6 and S7) or with different numbers of expressed genes (Additional file 2: Figures S8 and S9). Therefore, the number of genes or the species from which the data was produced is not a factor that affects clustering performance. However, the dispersion of clusters produced by all methods is dependent on the number conditions under consideration such that the more conditions being considered the worse the results of the clustering (Additional file 2: Figure S10). This is particularly problematic for cluster overlap (Additional file 2: Figure S11), where the clusters produced by all methods except *clust* become less distinct as the number of conditions increases.

### Clust outperforms other methods on seven different cluster validation indices

In order to provide an independent assessment of the performance of *clust*, the clusters produced by all of the 8 methods across all 100 datasets were assessed using seven additional cluster validation/separation indices. The indices comprise the Davies-Bouldin (DB) index [19], the Bayesian information criterion (BIC) [20], the silhouette index [21], the Calinski-Harabasz (CH) index [22], the Ball and Hall (BH) index [23], the Xu index [24], and the within-between (WB) index [25]. None of these seven validation indices are direct targets of optimization by *clust*. In contrast, some of the other tested clustering methods directly optimize some of these metrics (e.g., CC is designed to optimize the silhouette index [12]). To compare the methods on these metrics (whose scores differ in location shape and scale), a non-parametric rank-based comparison was performed. This revealed that *clust* significantly outperformed all of the other tested clustering methods (Fig. 3e; Additional file 9: Table S7). For example, *clust* shows significantly lower (better) rank scores than its closest competitor, CC, with a paired Wilcoxon test *p* value of 3.0 × 10^−15^.

**Fig. 4:**
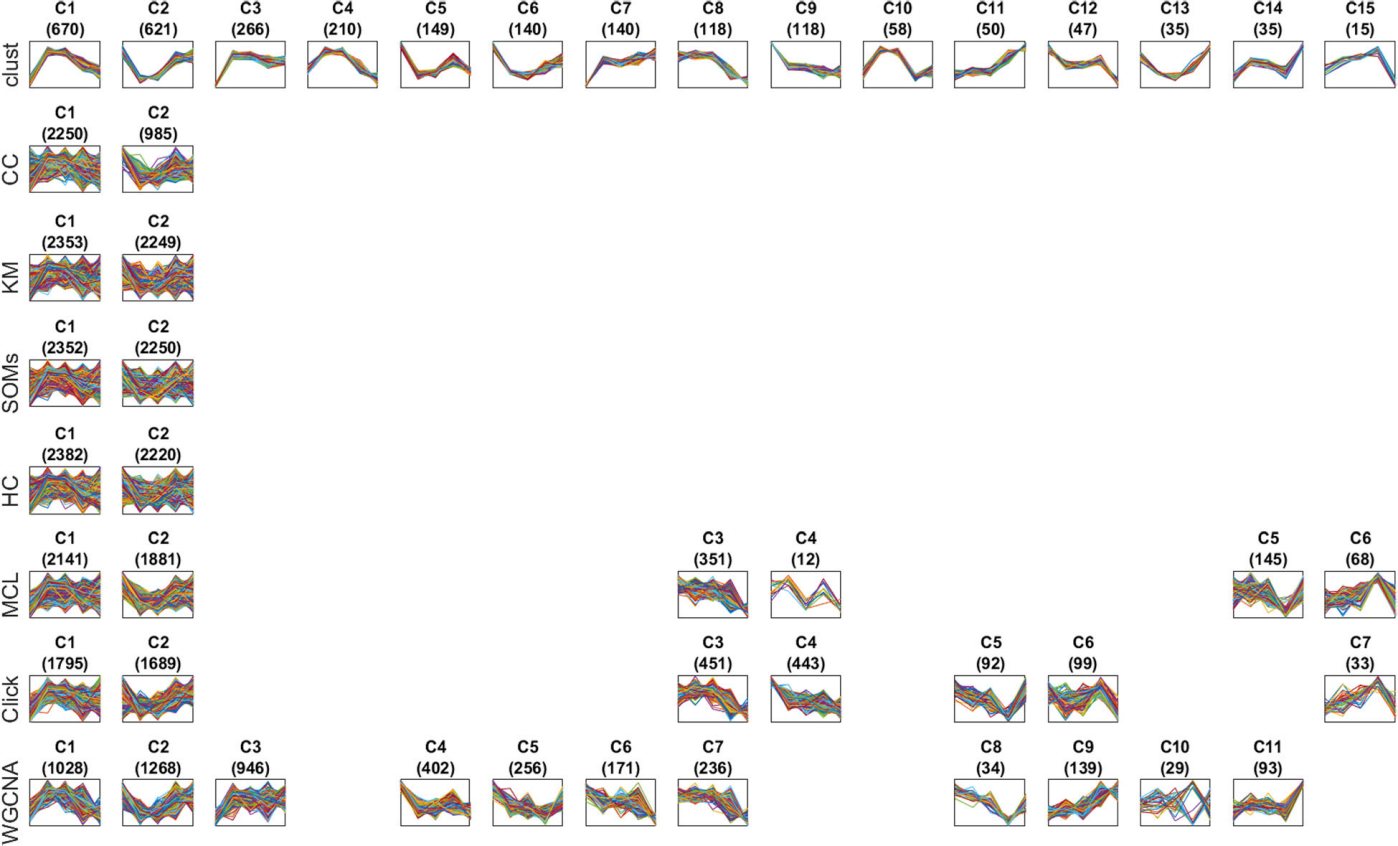
Profiles of the genes in the clusters generated by methods when applied to dataset D83. This figure visually shows a sample of the results of each one of the methods when applied over the same dataset, which is the dataset D83 (Additional files 4 and 6: Tables S2 and S4). This dataset is the time-series dataset of which the numbers of clusters generated by the eight methods are more similar to each other than any other time-series dataset (measured by the least squares metric). D83 is a budding yeast dataset with the accession GSE72423 and was generated using the Affymetrix Yeast Genome 2.0 microarray. Cells were grown in selective media supplemented with dextrose as a pre-culture and then shifted to media containing ethanol as the sole carbon source. Samples were taken at 0, 0.5, 1, 4, and 12 h after medium transfer. The numbers of clusters generated for this dataset by *clust*, CC, *k*-means, SOMs, HC, MCL, Click, and WGCNA were 15, 2, 2, 2, 2, 6, 7, and 11, respectively. This figure shows all 15 clusters generated by *clust* in the first row. Then, the most similar clusters generated by the other methods to each one of the 15 *clust*’s clusters are aligned below them. The title of each sub-plot shows the name of the cluster and the number of genes in that cluster between parentheses. The horizontal axis of each sub-plot represents the five time-points in the dataset D83 while the vertical axis represents the normalized gene expression value. The profiles of all individual genes in a cluster are drawn as lines on top of each other in its corresponding sub-plot

Figure 4 shows a comparative example of the clusters produced by each one of the eight clustering methods when applied to one of the 100 datasets, namely D83 (Additional files 4 and 6: Tables S2 and S4). This dataset was chosen as it is the time-series dataset with the most similar number of clusters across all the tested methods. A similar figure showing (up to) 14 clusters produced by each method for all 100 datasets is provided for download from the Zenodo repository at 10.5281/zenodo.1298541 [26]. The reduced MSE and JI of *clust* in comparison to other methods is readily apparent from visual inspection of the gene expression profiles of genes assigned to each cluster in Fig. 4.

### Clust extracts clusters with significantly enriched biological terms

One of the most commonly applied tests to co-expressed clusters of genes is functional term enrichment, as a cluster of co-expressed genes is expected to be enriched with genes that have related biological roles. As *clust* assigns on average 50% of genes to clusters, it was investigated if this reduction in gene number affects enrichment with functional terms. To do this, each of the methods were evaluated for their ability to detect enrichment of GO terms in 10 datasets from the multicellular organism *Arabidopsis thaliana*, and 10 datasets from the unicellular organism *Saccharomyces cerevisiae*.

**Fig. 5:**
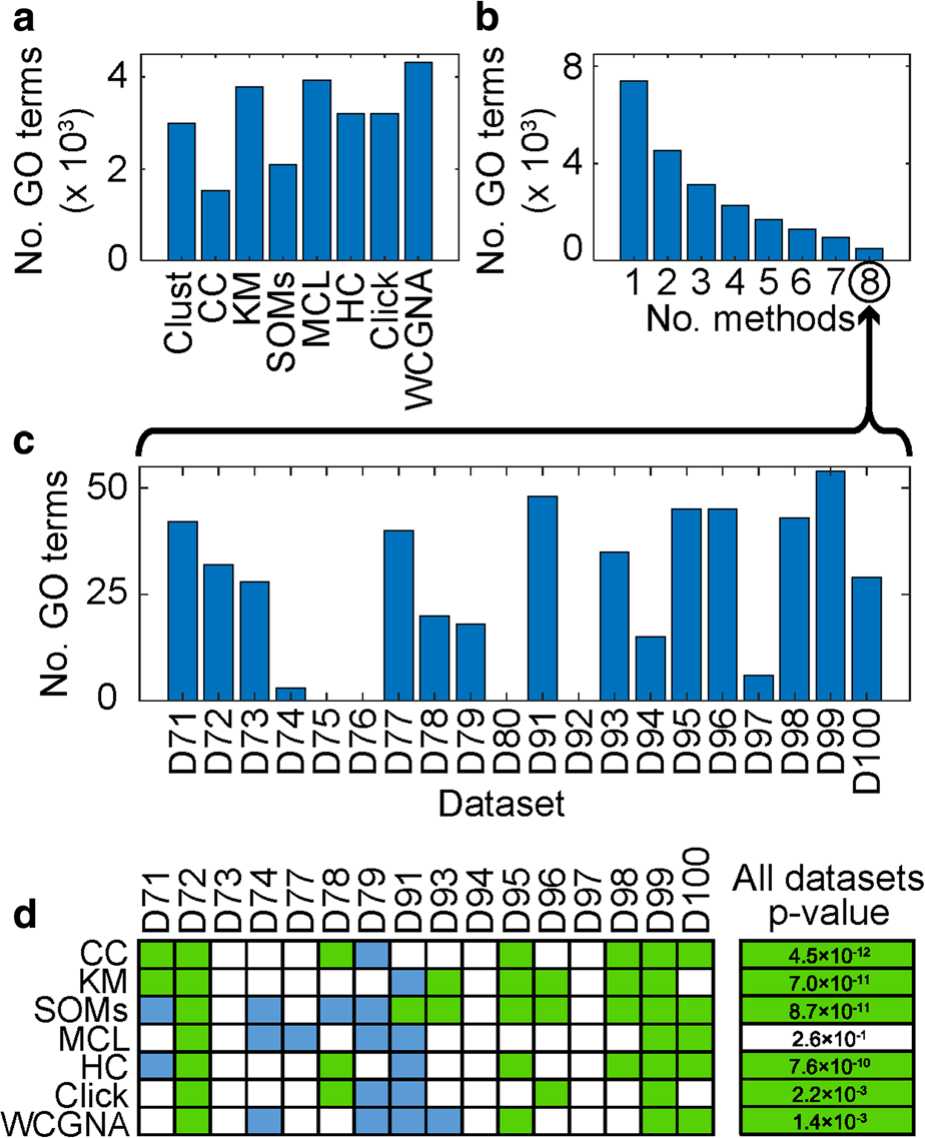
Evaluation of GO term enrichment in the results of the clustering methods. **a** The total numbers (sum) of GO terms detected as significantly enriched in the results of each of the eight methods across the 20 selected datasets. **b** Numbers of terms detected as significantly enriched in the same dataset by *x* or more methods; over the 20 datasets, 7404 terms were detected by at least one method, 2873 (39%) of which are exclusive to a single method, and only 503 (7%) terms were unanimously agreed by all eight methods. **c** The distribution of the 503 unanimously agreed GO terms over the 20 datasets. **d** Pairwise comparisons of the *p* values of the unanimously agreed GO terms in the clusters returned by *clust* with each of the other clustering methods. Green squares indicate that the *p* values for the GO terms returned by *clust* were better than those of the comparative method (Wilcoxon test *p* value ≤ 0.01), blue squares indicate the opposite result (Wilcoxon *p* value ≥ 0.99), and white squares indicate that there was no significant difference (0.01 < *p* < 0.99). The values to the right side of this matrix are the resultant *p* values when the Wilcoxon test is applied to the full dataset of all 503 unanimously agreed GO terms

Over the results of clustering these 20 datasets, different methods produced results with different numbers of enriched GO terms ranging from 1530 terms in the results of CC to 4317 terms in the results of WGCNA (Fig. 5a). *Clust*’s results include 2988 enriched GO terms (Fig. 5a). In total, 7404 GO terms were detected by at least one method, of which 4531 (61%) were detected by two or more methods and only 503 (7%) were detected by all methods (Fig. 5b; Additional file 10: Table S8). A similar observation can be seen when replicating this analysis using functional annotation terms from the REACTOME database [27] instead of GO terms (Additional file 2: Figure S12; Additional file 11: Table S6).

Given the disparity in detection of enriched functional terms between methods, and that the truth is unknown, a test was devised to assess which method best recovered those GO terms that were most likely to be true. Here the set of GO terms that were most likely to be true were defined as those that were identified as significantly over-represented within a given dataset by all methods (*n* = 503). The distribution of these 503 unanimously agreed terms in the different constituent datasets is shown in Fig. 5c. It should be noted here that such unanimous terms were not identified in four of these datasets (Fig. 5c) and thus only 16 of the 20 datasets contributed GO terms to this analysis. Although there was variation in the performance between methods on different datasets (Fig. 5d), overall, the clusters produced by *clust* achieved significantly better *p* values for these unanimous GO terms than clusters produced by CC, *k*-means, SOMs, HC, Click, or WGCNA, while being not significantly different from those produced by MCL (Fig. 5d; Additional file 10: Table S8; see the “Methods” section for description of the statistical tests). Thus in addition to improved clustering performance, as defined by multiple cluster validation indices, *clust* also performs as well as or better than other tested clustering methods in terms of GO term detection.

### Clust extracts clusters of co-expressed genes from multiple datasets simultaneously

The quantity of gene expression data that is deposited in public repositories is increasing rapidly. This is primarily due to a reduction in the costs of acquiring such datasets. These datasets come from a multitude of different species, have been generated using different technologies (microarrays and RNA-seq), and have different properties such as numbers of conditions, replicates, and missing values. *Clust* is designed to enable simultaneous cluster extraction from multiple such heterogeneous gene expression datasets (Additional file 3: Text S1). Irrespective of datatype or source species, *clust* extracts clusters of genes that are consistently co-expressed with each other in all of the given datasets.

To evaluate this feature of *clust*, ten combinations of *d* datasets for each *d* in {2,3,4,5,6,7,8,9,10} were selected at random from the ten yeast RNA-seq datasets (D91 to D100; Additional file 4: Table S2). The same experiment was performed over Arabidopsis datasets. To provide a comparison, the other methods were also applied to these combinations of datasets. However, as these methods are only applicable to a single dataset at a time, the only way to enable their simultaneous analysis was to concatenate them together prior to clustering (Additional files 12, 13, 14, 15: Tables S10–S13). As before, *clust* produces tighter clusters with lower within-cluster dispersion (lower MSE) (Additional file 2: Figure S13a & b) and guarantees no cluster profiles which overlap (JI = 0, Additional file 2: Figure S13c & d). Moreover, and as expected from a biological point of view, both the percentage of input genes that are included in the extracted clusters (PAG) and the number of generated clusters (*K*) decrease as more datasets are included as input to *clust* (Additional file 2: Figure S13e–h). This behavior is expected because as the number of conditions increases, the less likely a group of genes are to be co-expressed under all conditions. For example, when all ten yeast RNA-seq datasets are provided as input to *clust*, only a single cluster of 52 genes is identified. Of these, 44 are components of the ribosome or participate in ribosome biogenesis (Additional file 16: Table S14). An analogous cluster of 19 genes (all of them are involved in translation (protein synthesis)) was obtained when all 10 Arabidopsis RNA-seq datasets were provided to *clust* (Additional file 16: Table S14).

Of the other methods, MCL maintains relatively low MSE values over increasing numbers of datasets (*d*). In contrast, MSE values of the other methods increase when *d* increases (Additional file 2: Figure S13a–d). Moreover, MCL is the only method, other than *clust*, which shows the biologically expected trend of decreasing values of PAG and *K* at higher *d* values (Additional file 2: Figure S13e–h). Nonetheless, the performance of *clust* is significantly better than MCL in terms of MSE and JI values at all *d* values (Additional file 2: Figure S13a–d).

## Discussion and conclusions

Co-expression clustering is a routinely used step in data exploration for gene expression analysis. Here we show that the most commonly used methods for conducting co-expression analysis produce clusters that substantially disagree with each other and do not match the biological expectations of co-expressed clusters of genes. That is, they produce clusters that are highly dispersed (high MSE values) and contain large proportions of genes that could be equally assigned to other clusters within the same clustering result (high JI values). Moreover, the methods behave inconsistently, with substantial differences in clustering performance attributable to differences in datatype or data quantity. We present *clust*, as a method designed to solve all of these problems. *Clust* was compared with seven commonly used clustering methods (CC, *k*-means, SOMs, MCL, HC, Click, and WGCNA) by application to 100 different microarray and RNA-seq gene expression datasets from five model species. In contrast to the other tested methods, *clust* behavior is consistent and is unaffected by species, datatype, or number of genes over the diverse sample of 100 datasets analyzed in this study. Thus, *clust* performance is robust to increases in data quantity without sacrificing the quality of the results. Moreover, *clust* outperforms all of the tested methods when assessed by 7 commonly used cluster validation metrics.

The most commonly conducted post-clustering analysis is to detect enrichment of functional terms within clustered sets of co-expressed genes. We show that conducting such analyses on clusters produced using the most commonly used methods for co-expressed gene clustering produces very different results (Fig. 5b). This observation has implications for the utility of downstream analysis conducted on these clusters. For example, putative regulatory relationships are often inferred by identifying regulatory genes that occur in clusters that are enriched with specific functional terms [28–30]. Thus, unreliability of enriched functional term assignment likely contributes to the high false-positive discovery rate in the discovery rate of regulatory interactions from co-expression data [31]. We have shown that *clust* extracts clusters that are significantly more enriched with functional annotation terms than many other commonly used methods. Thus, *clust* not only outperforms other methods in terms of numerical properties of the clusters it produces but also produces the highest quality functional annotation term enrichment.

Finally, *clust* is designed to be able to extract clusters of co-expressed genes from multiple gene expression datasets, even if these datasets have different properties such as numbers of conditions or replicates. Such feature allows researchers who have multiple gene expression datasets that are all related to the biological problem in hand to analyze them simultaneously, that is, to extract the clusters of genes which are consistently co-expressed in each of these different datasets. Various consequences can be inferred from such analysis. For instance, it is more reliable to hypothesize that a group of genes are co-regulated by common regulator when they are consistently co-expressed over multiple datasets in contrast to being co-expressed in a single dataset only [2, 32–35]. Moreover, the results of applying *clust* to multiple datasets simultaneously are as biologically expected; fewer genes are included in the clusters when adding more datasets as fewer genes are expected to maintain coordinate expression over larger numbers of biological conditions.

Taken together, this work reveals a mismatch between what researchers expect from gene expression clustering and the results that are produced by application of commonly used data partitioning methods to these data. The proposed *clust* method solves this problem, and the utility and performance characteristics of *clust* are demonstrated through comprehensive testing and comparison on real biological datasets from multiple different species. In addition to improved performance characteristics over competing methods, the ability of *clust* to handle multiple datasets simultaneously will enable individual gene expression datasets to be interpreted in the context of the large quantity of publicly available gene expression data. *Clust* is open source and freely available at https://github.com/BaselAbujamous/clust [15].

## Methods

### Simulated gene expression data generation

Four simulated datasets were generated to allow visualizing the concept of gene expression clustering (Fig. 1). Each dataset is composed of gene expression profiles of 500 genes, 300 of which belong to three clusters with 100 genes in each, and 200 of which do not belong to any cluster (unclustered). The gene expression profiles span six simulated time-points over which the first cluster has an upregulated pattern, the second cluster has a downregulated pattern, the third cluster has an upregulated then downregulated pattern, and the unclustered genes have other random patterns. The 100 gene expression profiles in a given cluster are generated by adding random Gaussian noise to the average profile of that cluster. Four different standard deviation (σ) values of the Gaussian noise were considered for the four datasets such as the first dataset (D1) has a zero σ value and the last dataset (D4) has the highest σ value (σ = 1.2). The 200 unclustered genes were generated randomly using the Gaussian distribution while guaranteeing that none of them fits within the profiles of any of the three clusters. Finally, the datasets were normalized by calculating *z*-scores. Full expression values and cluster membership for these datasets are provided in Additional file 1: Table S1.

### Selection of 100 gene expression datasets

The 100 gene expression datasets were downloaded from the Gene Expression Omnibus (GEO) repository on 2nd of July 2017 [36]. For each one of the five model species, ten microarray datasets and ten RNA-seq datasets were downloaded. In all cases, the most recently published datasets for each of these species was selected, given that the dataset had at least 4 different conditions (time-points or treatments) and no more than 50 samples including replicates. RNA-seq datasets were chosen only if the resulting TPM, RPKM, FPKM, or CPM quantitation files were available from the GEO repository. Microarray datasets were a mix of both one-colour or two-colour microarrays. The complete list of the 100 datasets and their properties is available in Additional file 4: Table S2. The raw data files for the 100 datasets, the clustering from each method, and the analysis scripts are all publically available at the Zenodo repository with the doi 10.5281/zenodo.1298541 [26].

### Data pre-processing and the implementation of clust and comparative methods

All methods, including *clust*, were run using their default parameters, which is the manner in which they are most commonly used. The datasets were pre-processed according to the common practice of applying quantile normalization, taking the logarithm of data values (unless already taken in the downloaded data), and calculating *z*-scores. However, centring around zero was adopted instead of *z*-scores for two-colour microarray datasets. After that, replicates of the same condition were summarized by taking their median value. In addition, genes that do not exceed the 25th percentile expression value at least at 25% of the conditions/samples are filtered out. All of these pre-processing actions were carried out during the first step of *clust* before applying the following clustering steps. The *clust* software package is provided with parameters indicating these choices of pre-processing actions, and in its turn, it provides the pre-processed dataset in one of its output files. Consequently, the other methods were directly applied to these pre-processed data files.

The Python package *clust 1.8.0* was used to run *clust* [15]. The CC method was run using the R *CrossClustering* library. *K*-means was run using the Python *sklearn.cluster* implementation. The Python *sompy* package was used to run SOMs. The Python *scipy.cluster.hierarchy* package was used to run HC clustering. The Python *mcl* package was used to run MCL after generating networks of co-expressed genes using a Pearson’s correlation threshold of 0.8 [16]. The *click.exe* executable was downloaded as part of the *Expander* software from http://acgt.cs.tau.ac.il/expander/ and was used to run Click. The *blockwiseModules* module of the R *WGCNA* library was used to run WGCNA with the network type set to “signed”.

Running *k*-means, HC, and SOMs, requires pre-setting the number of clusters (*k*). Each of these methods was applied to the input data with *k* values ranging from 2 to 50 and the *k* value that minimized the Davies–Bouldin (DB) cluster validation index was chosen. The DB index is a widely used and frequently cited whole-partition cluster validation index [19]. To demonstrate that the superior performance characteristics of *clust* were not due to use of the DB index, we also attempted to bias against our principle finding by choosing the cluster sets that minimized our evaluation criteria, i.e., that minimized MSE and the JI metrics (minimizing $\sqrt{MSE^2+{JI}^2}$). These additional results are analogous to those produced using DB index and are included in Additional file 2: Figure S2 and Additional file 6: Table S4(B).

### Cluster dispersion metric (MSE)

The mean squared error (MSE) metric is used to measure within-cluster dispersion. If the cluster has *N* genes and the dataset has *D* dimensions, the MSE value for that cluster will be:

$$ MSE=\frac{1}{D\times N}\sum \limits_{g=1}^N{\left\Vert {\overrightarrow{x}}_g-\overrightarrow{z}\right\Vert}^2, $$

where ${\overrightarrow{x}}_g$ is a vector of the gene expression profile of the *g*^*th*^ gene in this cluster, $\overrightarrow{z}$ is a vector of the average expression profile of all genes in this cluster, and $\left\Vert {\overrightarrow{x}}_g-\overrightarrow{z}\right\Vert$ is the Euclidean distance between these two vectors. Note that the MSE value here is normalized by the number of genes in the cluster. When calculating the MSE value for a whole clustering result (a set of clusters), it is calculated as the weighted average of the MSE values of the each of the clusters, where the weight is the size (number of genes) in each of the clusters.

### Cluster similarity metric (JI)

A modified version of the Jaccard Index (JI) metric is used to measure the similarity amongst the clusters in a clustering result [37]. JI, as defined in this study, is calculated as the ratio between the number of “overlap genes” and the number of all genes in clusters. “Overlap genes” are those genes that are included in a cluster while their expression profiles also fit within the boundaries of at least one other cluster. The upper and the lower boundaries of a cluster at any given dimension (condition) are respectively calculated as the maximum and the minimum expression values of all genes in that cluster after trimming the most extreme 1% values at each point to reduce the effect of outliers.

### Cluster validation indices and the rank score

Each clustering result produced by applying a single clustering method to a single dataset is assessed by using seven cluster validation indices other than the MSE and the JI metrics. The indices are the Davies-Bouldin (DB) index [19], the Bayesian information criterion (BIC) [20], the silhouette index [21], the Calinski-Harabasz (CH) index [22], the Ball and Hall (BH) index [23], the Xu index [24], and the within-between (WB) index [25]. Larger values of the BIC, silhouette, and CH indices indicate better clustering results while smaller values of the DB, BH, Xu, and WB indices indicate better clusters.

Then, the absolute values of the indices are converted to rank scores where a clustering method’s rank score of a given index at a given dataset is 1.0 if that method scores the best index score across the eight clustering methods at that dataset and is 8.0 if it scores the worst index. If two or methods have the same score, their ranks are averaged (e.g., two methods sharing the best score across eight will have rank scores of 1.5 for each). Therefore, these seven rank scores reflect how well the method behaves in comparison with the other clustering methods while clustering that dataset. After that, the seven rank scores for each method at each dataset are averaged to arrive at the final rank score for that method at that dataset. The final scores (plotted in Fig. 3e) are 100 scores for each method reflecting its rank across the 100 datasets.

### GO term enrichment analysis

The GO term annotations for *Arabidopsis thaliana* and *Saccharomyces cerevisiae* were downloaded from the Gene Ontology Consortium’s online repository at http://www.geneontology.org [38, 39]. Clusters produced using each method were evaluated on a dataset by dataset basis (i.e., 20 independent datasets). Significantly over-represented GO terms for each dataset for each method were taken as the set of terms that each obtained an adjusted hypergeometric test *p* value ≤ 0.001. The full set of these terms for each method and dataset are provided in Additional file 10: Table S8.

To enable direct comparison between methods, a set of unanimously agreed GO terms was identified. A unanimously agreed GO term is defined here as a GO term that was detected as significantly over-represented (*p* value ≤ 0.001) by all methods in a given dataset. GO terms from each dataset were treated independently irrespective of whether they were also observed as significantly over-represented in other datasets, i.e., the *p* value for GO term 1 from dataset 1 was not compared to the *p* value for the same GO term in a different dataset. In the case where the same GO term was detected multiple times within a single method clustering of a single dataset, then the instance with the lowest *p* value was taken for analysis.

A paired one-tailed Wilcoxon test was used to compare log transformed *p* values of the unanimously agreed GO terms between methods. This pairwise comparison was conducted for each dataset separately (Fig. 5d), as well as for the full set of unanimously agreed GO terms combined from all datasets (Fig. 5d).

## Additional files

Additional file 1: **Table S1.** Full expression values and cluster membership of the simulated gene expression datasets in Fig. 1. (XLSX 197 kb)

Additional file 2: **Figures S1 to S13.** Manuscript Supplementary Figures S1 to S13. (PDF 931 kb)

Additional file 3: **Text S1.** The full description of the *clust* algorithm. (PDF 353 kb)

Additional file 4: **Table S2.** A list of the 100 gene expression datasets analyzed in this study. (XLSX 18 kb)

Additional file 5: **Table S3.** Adjusted rand index (ARI) scores measuring the similarity in cluster membership between the results of every pair of methods when applied to each of the 100 datasets. (XLSX 31 kb)

Additional file 6: **Table S4.** MSE and JI values for whole clustering results as generated by each of the eight methods when applied to each of the 100 datasets. (XLSX 53 kb)

Additional file 7: **Table S5.** A list of the 19 gene expression datasets which have more samples than 50 (complementary list to the 100 datasets list). (XLSX 14 kb)

Additional file 8: **Table S6.** MSE and JI values for all individual clusters generated by each of the eight methods when applied to each of the 100 datasets. (XLSX 335 kb)

Additional file 9: **Table S7.** Values of each one of the seven cluster validation indices DB, BIC, Silhouette, CH, BH, XU, and WB, and the rank scores, when calculated for the results of applying each one of the eight clustering methods to each of the 100 datasets. (XLSX 60 kb)

Additional file 10: **Table S8.** Lists of GO terms enriched in whole clustering results generated by each of the eight methods when applied to each of the datasets. (XLSX 1051 kb)

Additional file 11: **Table S9.** Lists of REACTOME pathways enriched in whole clustering results generated by each of the eight methods when applied to each of the datasets. (XLSX 282 kb)

Additional file 12: **Table S10.** MSE values for the results of applying each of the eight clustering methods to multiple datasets simultaneously. (XLSX 134 kb)

Additional file 13 **Table S11.** JI values for the results of applying each of the eight clustering methods to multiple datasets simultaneously. (XLSX 133 kb)

Additional file 14: **Table S12.** Percentages of genes included in the clusters in the results of applying each of the eight clustering methods to multiple datasets simultaneously. (XLSX 149 kb)

Additional file 15: **Table S13.** Numbers of clusters generated by applying each of the eight clustering methods to multiple datasets simultaneously. (XLSX 130 kb)

Additional file 16: **Table S14.** GO terms of some of the results of applying *clust* to multiple datasets simultaneously. (XLSX 17 kb)
