# Supplementary material for: Clust: automatic extraction of optimal co-expressed gene clusters from gene expression data
Source: Genome Biol. 2018 Oct 25;19:172. doi: 10.1186/s13059-018-1536-8 (PMC6203272; doi:10.1186/s13059-018-1536-8)
Supplement: Supplementary file 2 — Figures S1 to S13. Manuscript Supplementary Figures S1 to S13. (PDF 931 kb) [file 13059_2018_1536_MOESM2_ESM.pdf]

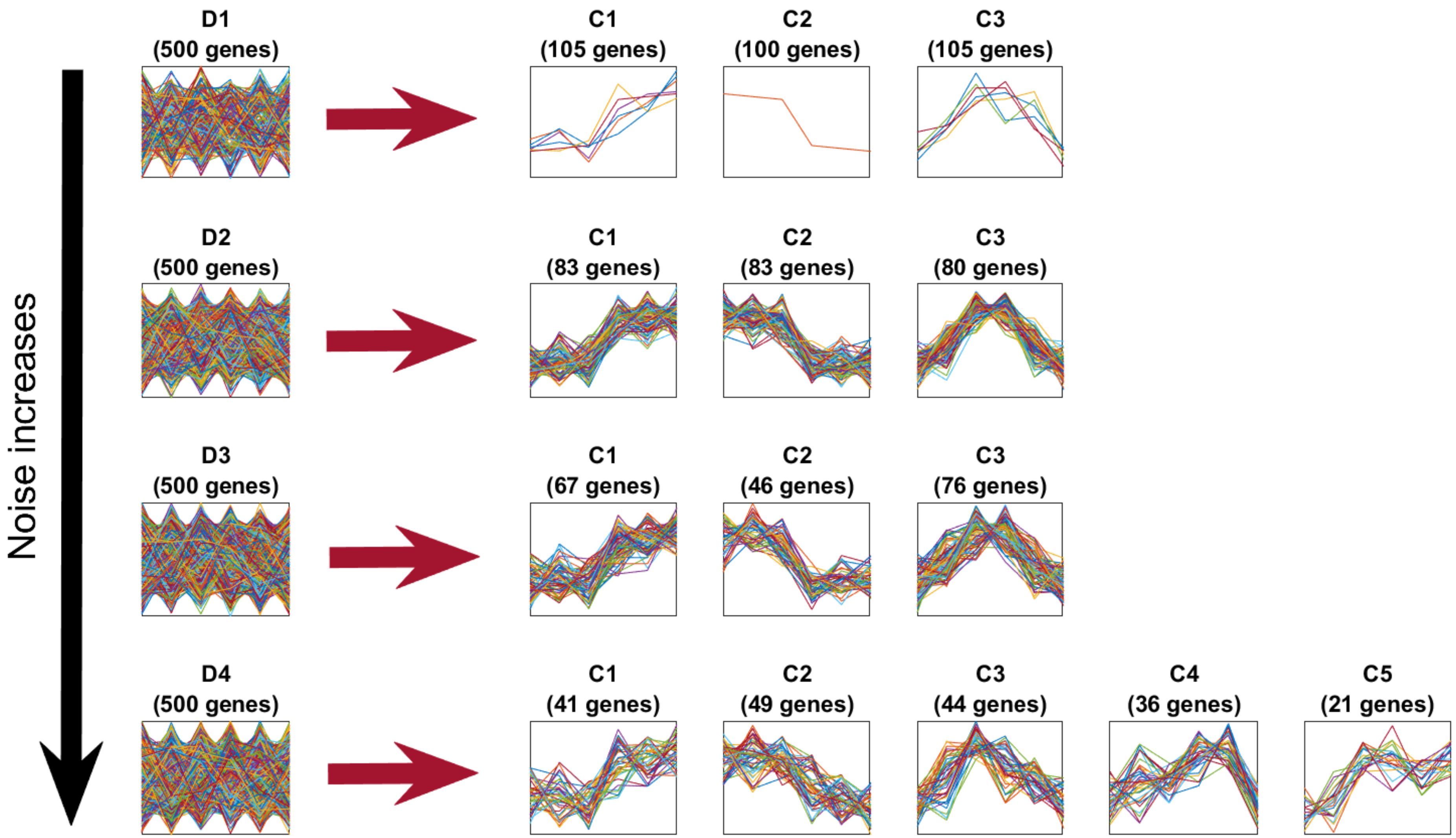

Figure S1. Results of applying *clust* to the simulated datasets described in main paper Fig. 1.
Left panel: all of the 500 gene expression profiles in each one of the four simulated datasets. Right panel: the clusters that *clust* extracted from each one of these datasets.

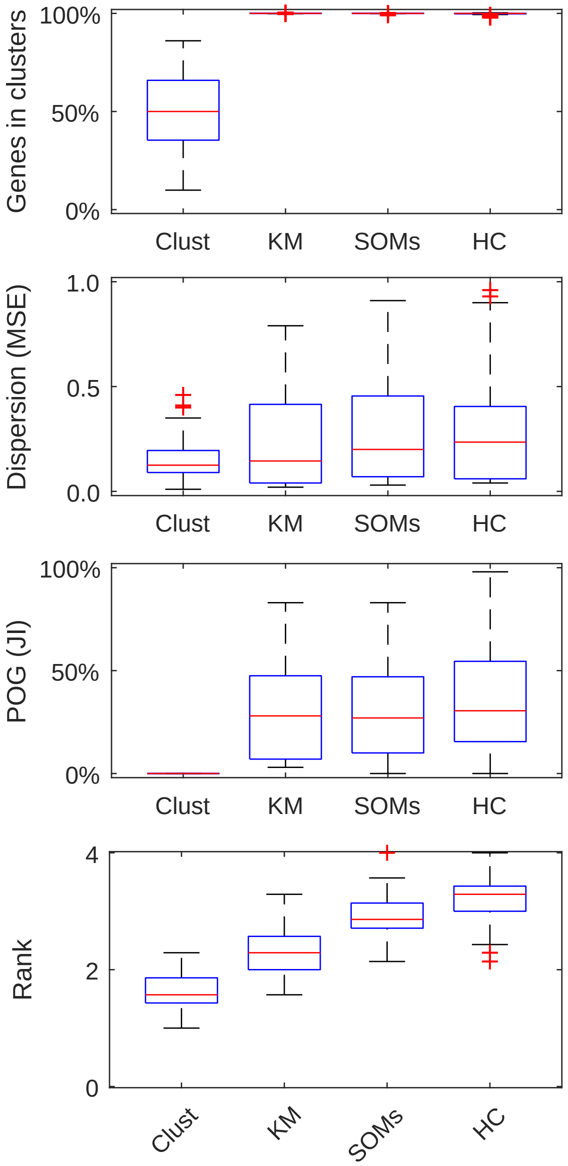

This is similar to Fig. 3 (b,c,d,h) in the main paper.

The clustering methods k-means (KM), SOMs, and hierarchical clustering (HC) require pre-setting the number of clusters (k). They were applied to each one of the 100 datasets with all k values from 2 to 50 and then the result at the k value with the best quality is adopted for the corresponding dataset. In Fig. 3 in the main paper, the best result is defined as the one which minimses the commonly used DB cluster validation index. Here, it is defined as the one which miminses the MSE and the JI values. More specifically, by minimising sqrt(MSE^2 + JI^2).

CC, MCL, Click, and WGCNA automatically identify the number of clusters, and therefore this experiment is not applicable to them.

Using the paired t-test, the MSE values produced by clust are significantly lower than those of KM (p-value 2.7×10^{-5}), SOMs (p-value 1.8×10^{-9}), and HC (p-value 2.8×10^{-9}). As for the JI metric, clust guarrantees a zero JI, while the JI for the three other methods ranges from 10% to 50%. Rank over the seven utilised cluster separation mindices shows clust as clearly significantly better than the three other methods; for instance, paired t-test p-value with the closest competitor (KM) is 5.6×10^{-20}.

Figure S2. Comparing *clust* with *k*-means, SOMs, and HC when they are optimised based on the MSE and the JI metrics.

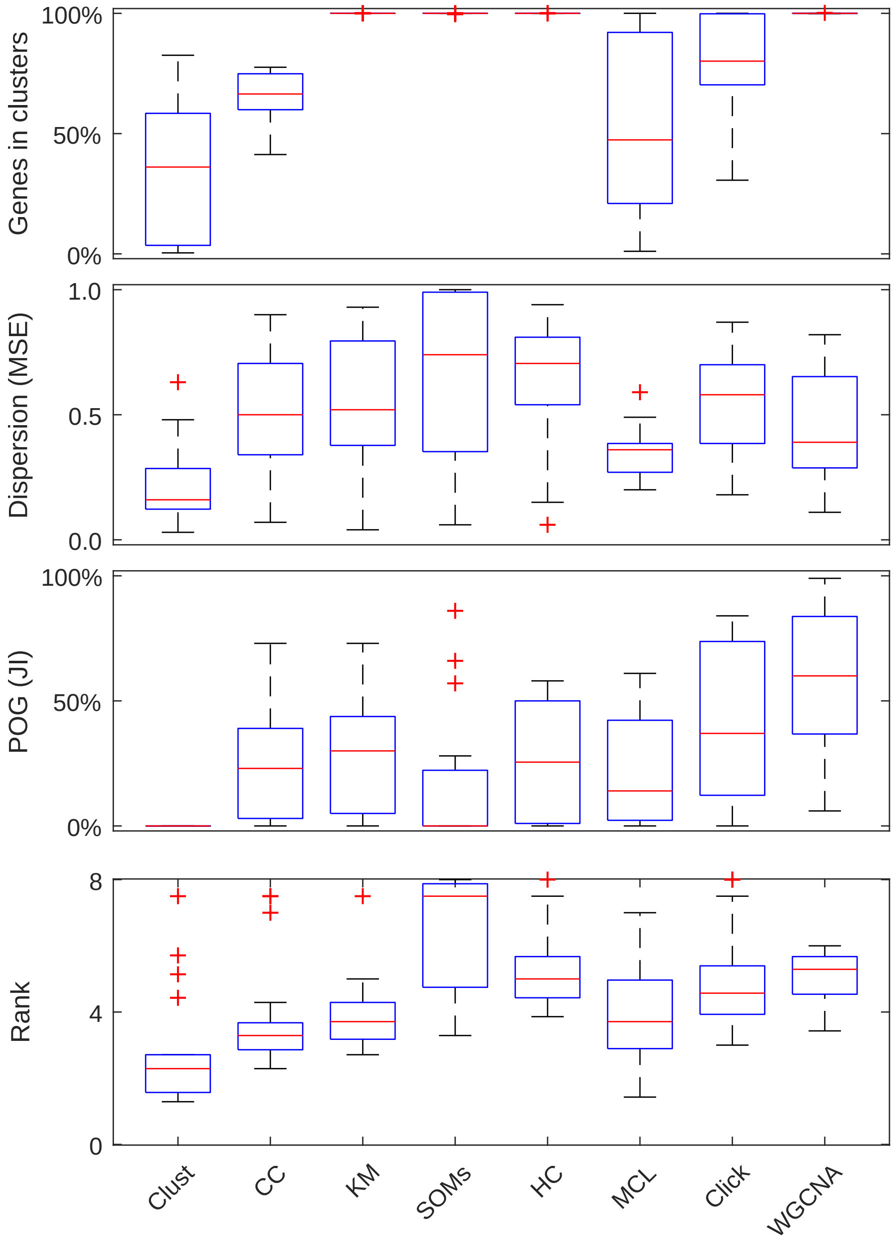

Figure S3. Large datasets clustering analysis

This is similar to Fig. 3a,b,c,h in the main text, but while analysing 19 large datasets (other than the 100 datasets analysed in the main paper). Large datasets are those with more than 50 samples (including replicates).

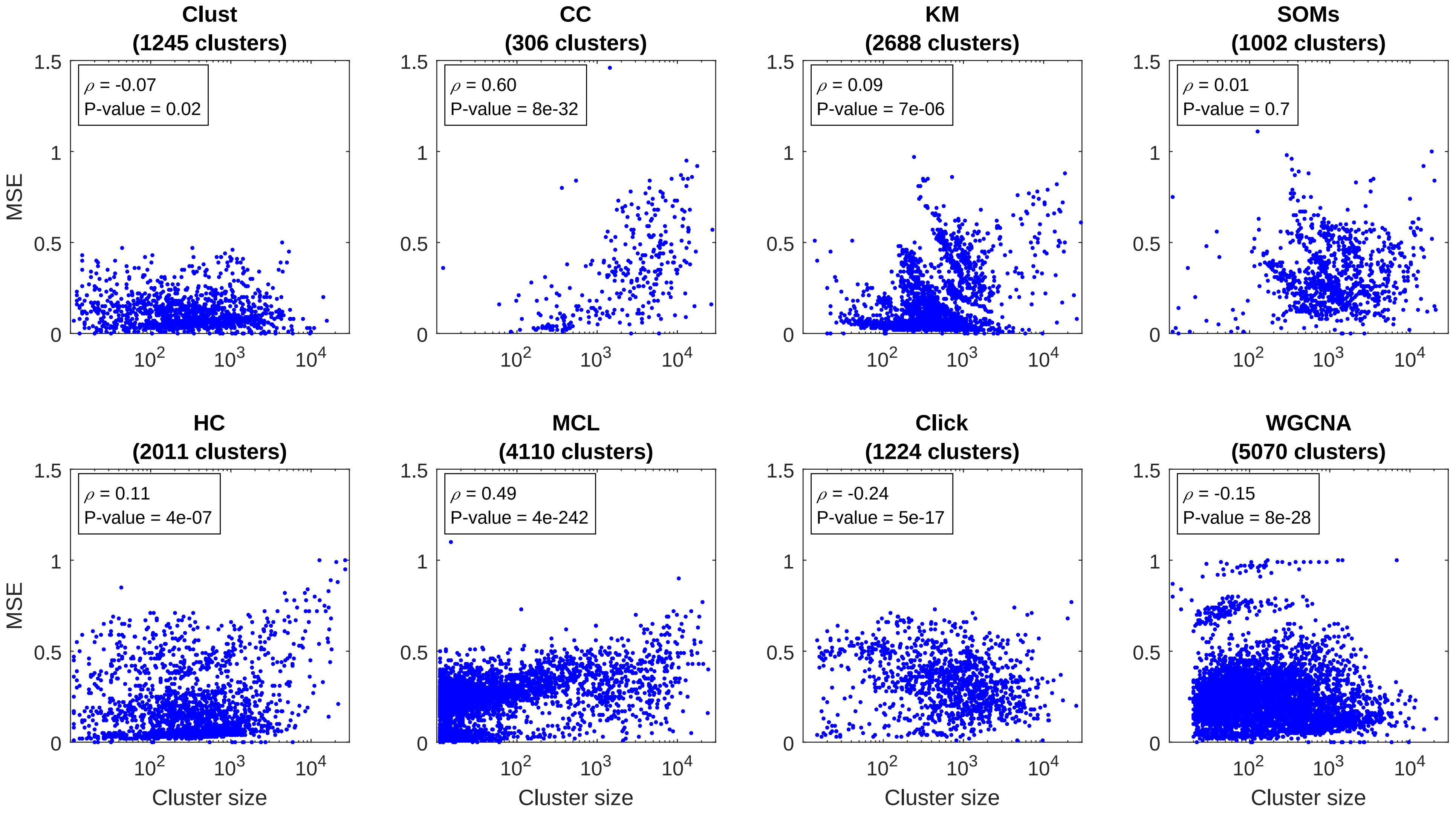

Figure S4 - MSE versus cluster size analysis

All individual clusters generated by each method over all of the 100 datasets are plotted here; every cluster as one point. Each plot shows cluster dispersion, measured by MSE, on the vertical axis, and cluster size (number of genes in the cluster) on the log-scaled horizontal axis. Spearman's correlation values (ρ) between MSE and cluster size are shown alongside their associated p-values.

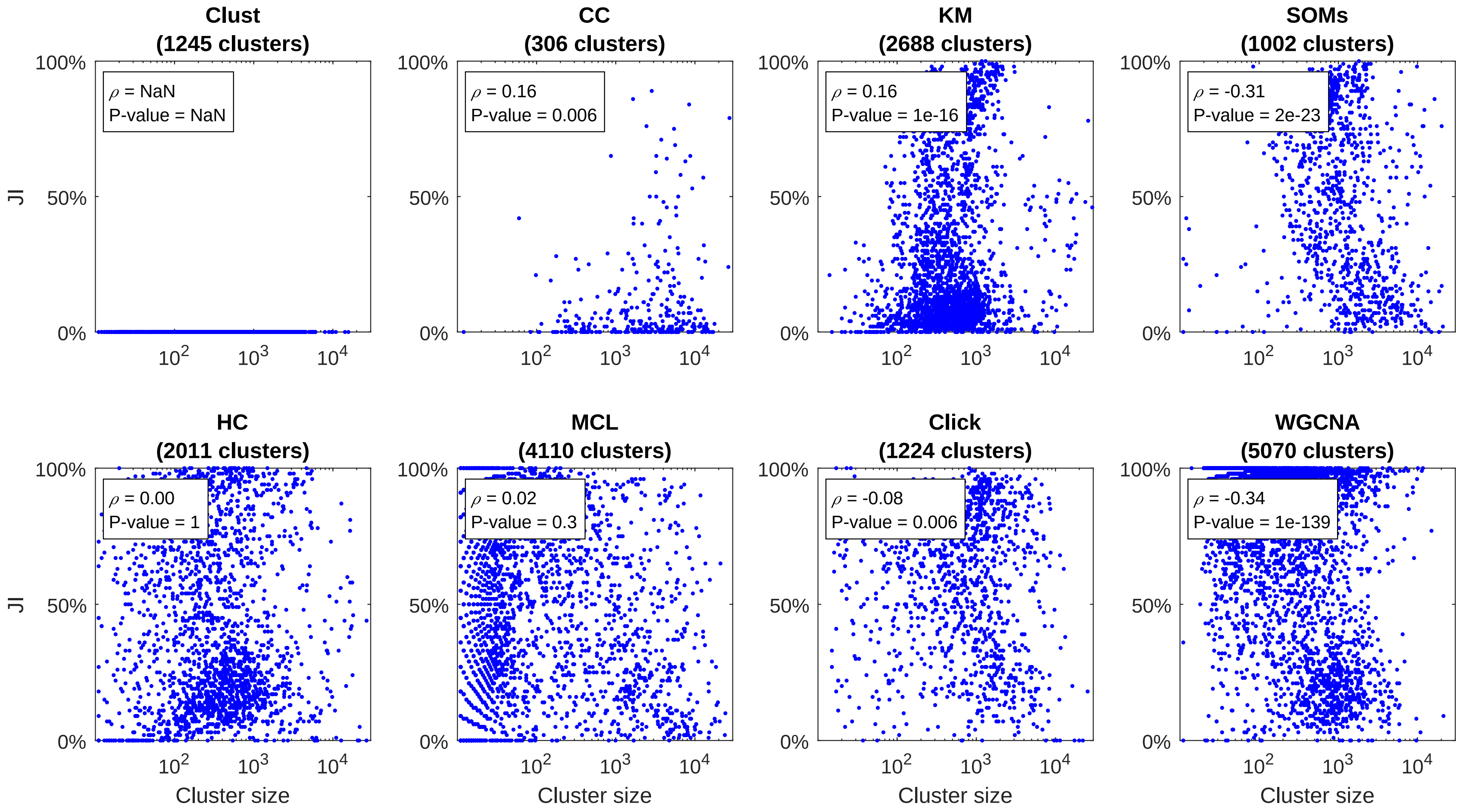

Figure S5 - JI versus cluster size analysis

All individual clusters generated by each method over all of the 100 datasets are plotted here; every cluster as one point. Each plot shows the percentage of cluster overlap (POG), measured by JI, on the vertical axis, and cluster size (number of genes in the cluster) on the log-scaled horizontal axis. Spearman's correlation values (ρ) between JI and cluster size are shown alongside their associated p-values.

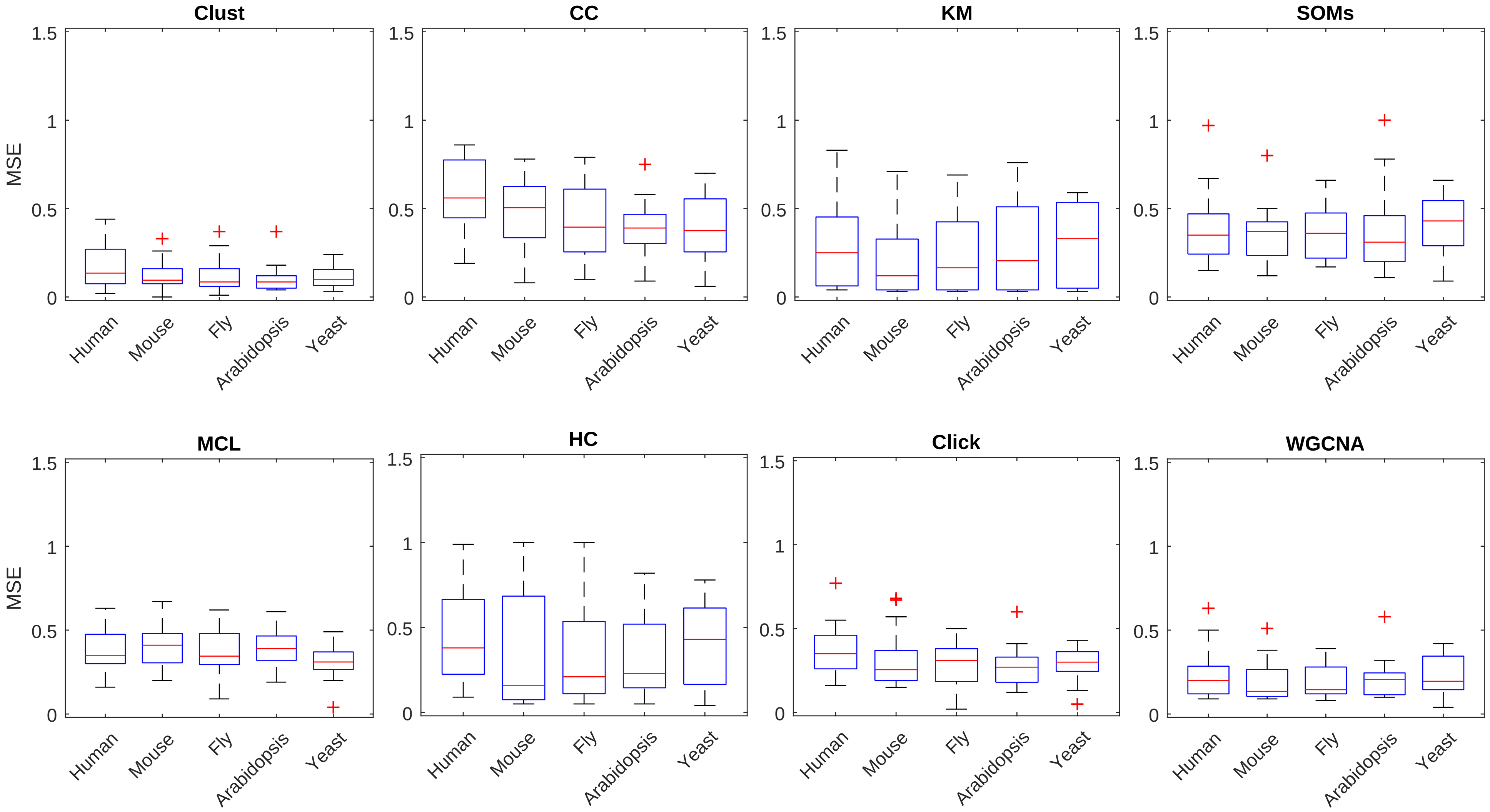

Figure S6 - MSE versus species analysis

Summary MSE values per dataset are plotted in these boxplots grouped by species. Each box includes MSE values of the results of applying the corresponding method to each one of the 20 datasets from the corresponding species.

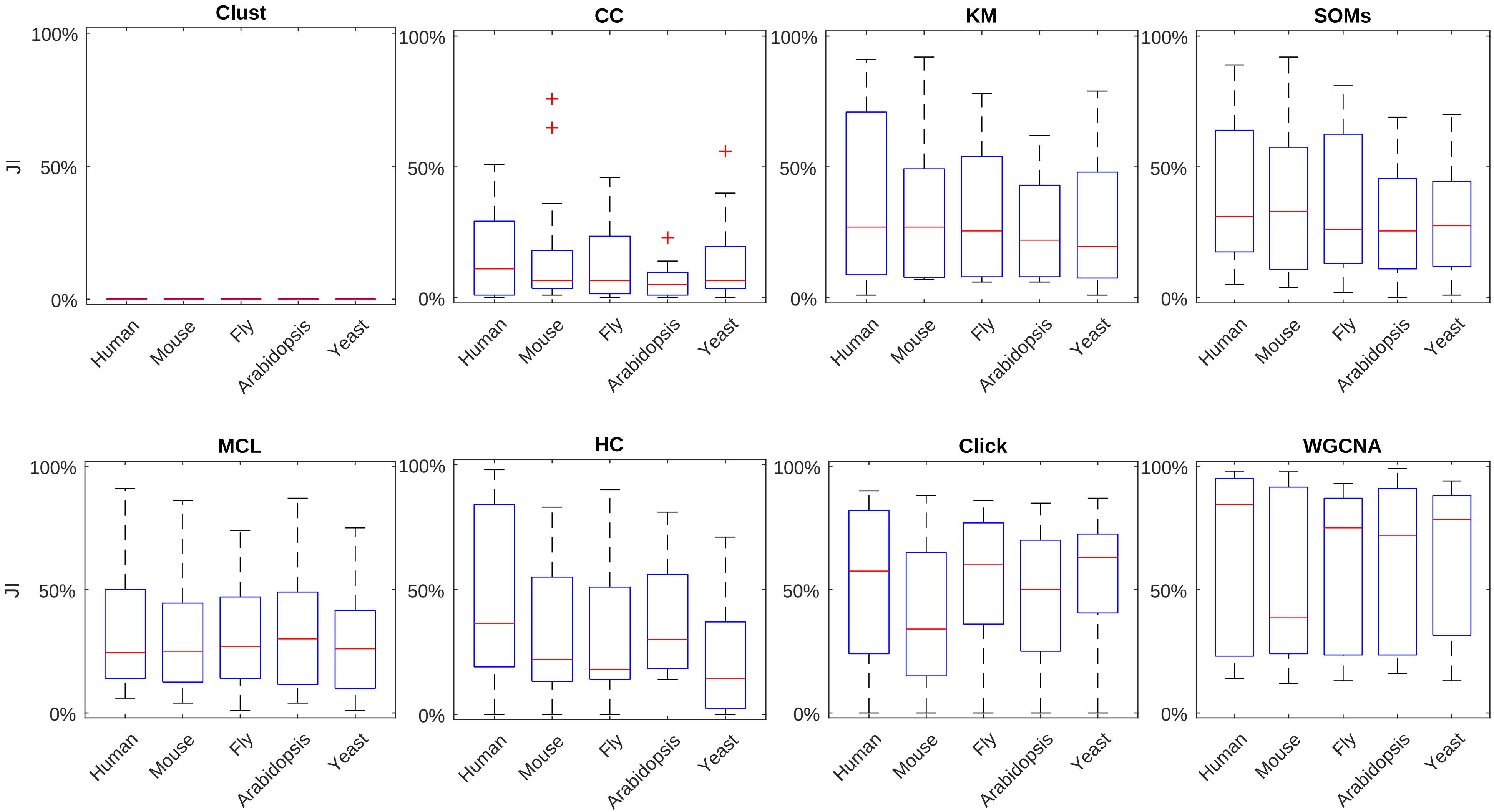

Figure S7 - JI versus species analysis

Summary MSE values per dataset are plotted in these boxplots grouped by species. Each box includes JI values of the results of applying the corresponding method to each one of the 20 datasets from the corresponding species.

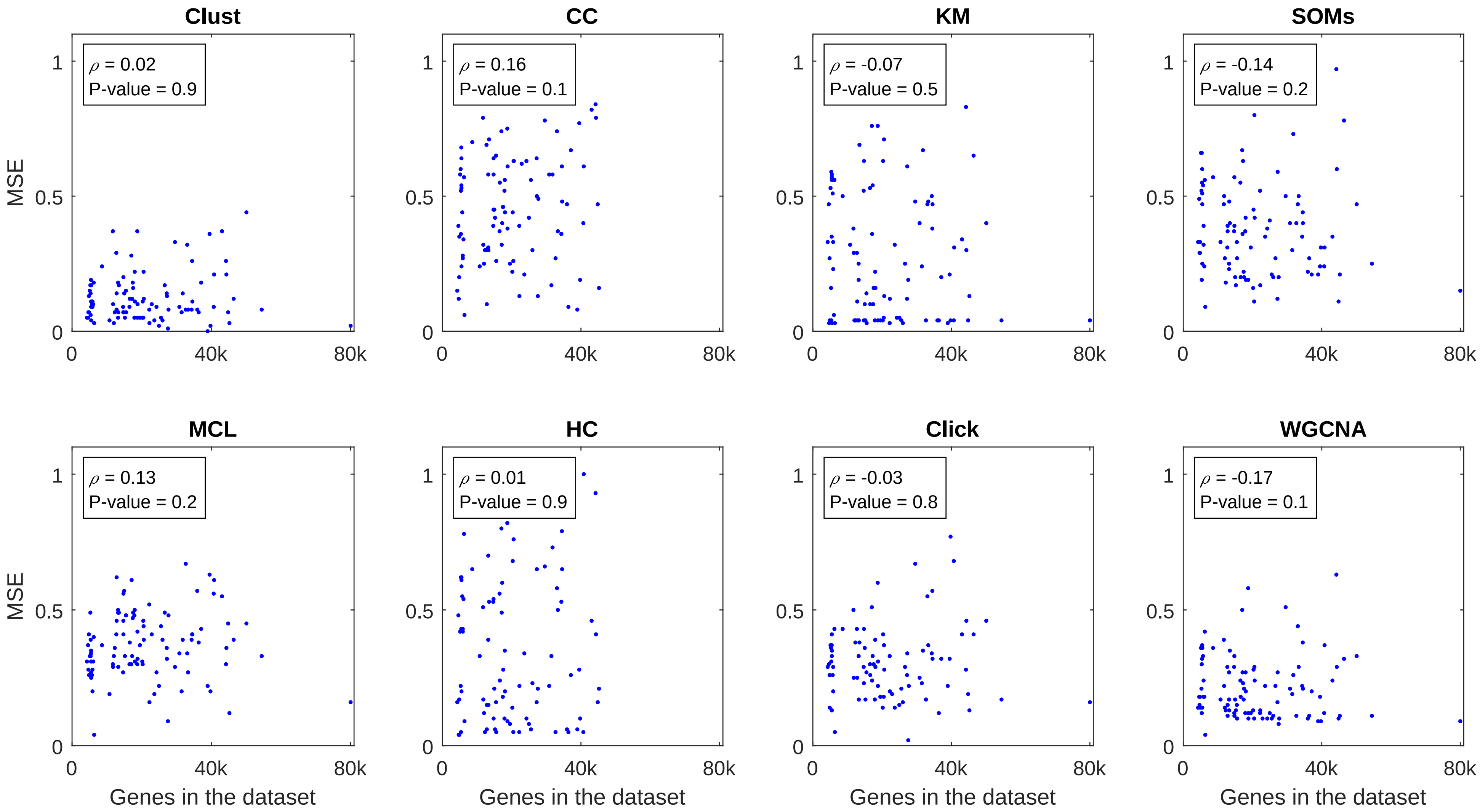

Figure S8 - MSE versus number of genes in the dataset analysis

Each scatter plot shows 100 points corresponding to the clustering results of applying the named method to each one of the 100 datasets. That is, each point represents one whole clustering result. Each plot shows average cluster dispersion, measured by MSE, on the vertical axis, and the number of genes expressed in the dataset on the horizontal axis. Spearman's correlation values (ρ) between MSE and the number of genes in the dataset are shown alongside their associated p-values.

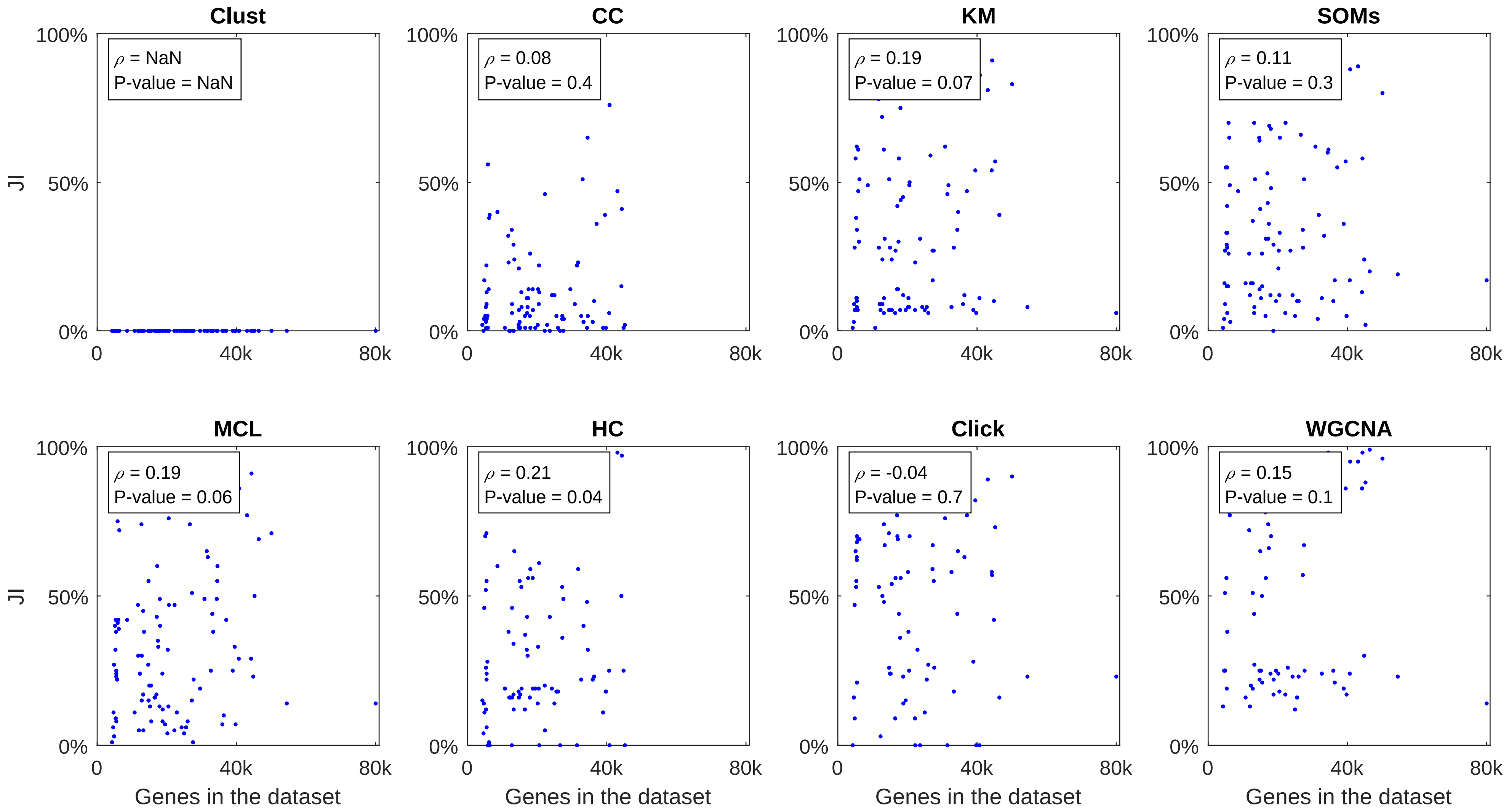

Figure S9 - JI versus number of genes in the dataset analysis

Each scatter plot shows 100 points corresponding to the clustering results of applying the named method to each one of the 100 datasets. That is, each point represents one whole clustering result. Each plot shows average percentage of cluster overlap (POG), measured by JI, on the vertical axis, and the number of genes expressed in the dataset on the horizontal axis. Spearman's correlation values (ρ) between JI and the number of genes in the dataset are shown alongside their associated p-values.

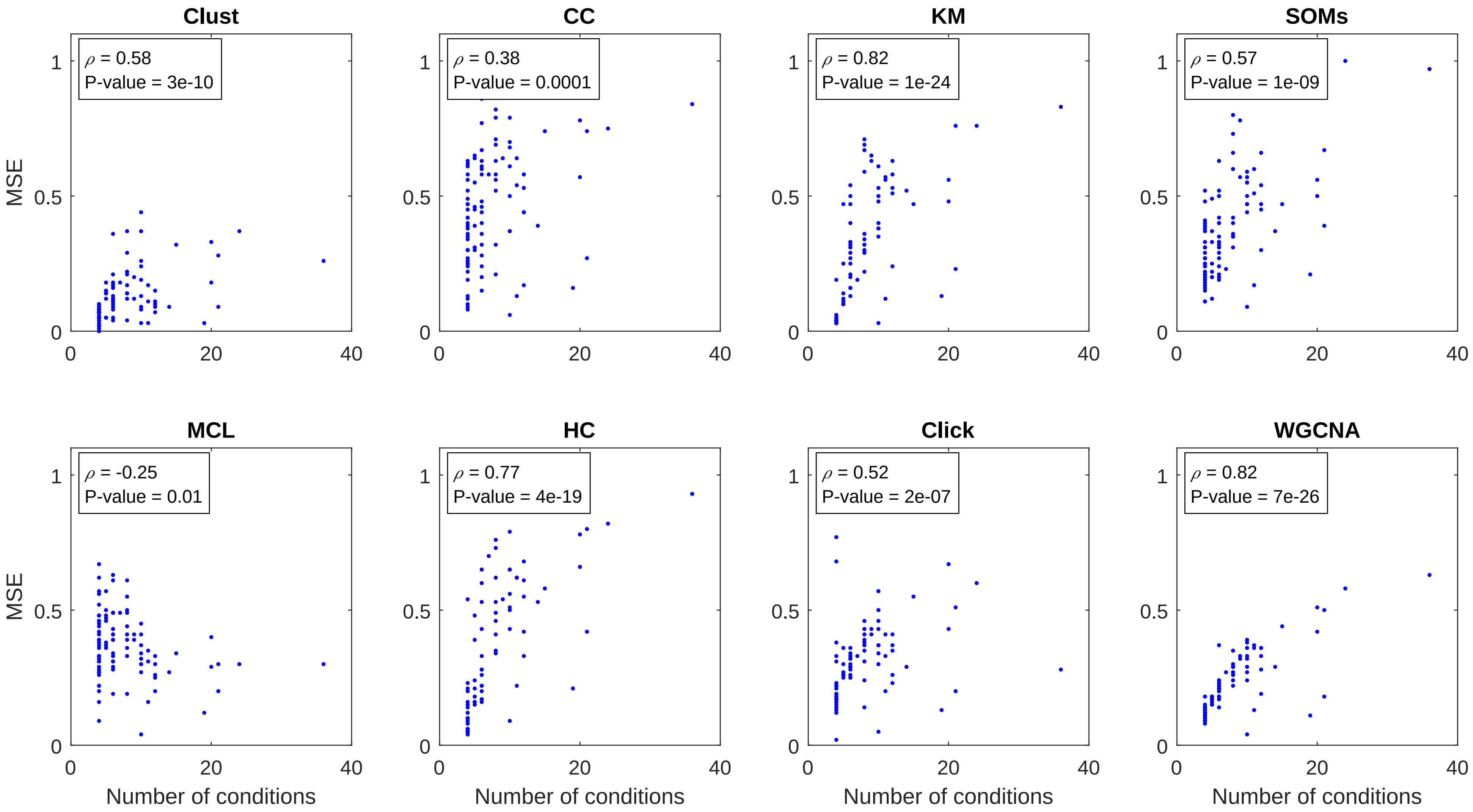

Figure S10 - MSE versus number of conditions (samples) in the dataset analysis

Each scatter plot shows 100 points corresponding to the clustering results of applying the named method to each one of the 100 datasets. That is, each point represents one whole clustering result. Each plot shows average cluster dispersion, measured by MSE, on the vertical axis, and the number of conditions (samples) in the dataset on the horizontal axis. Spearman's correlation values (ρ) between MSE and the number of conditions in the dataset are shown alongside their associated p-values.

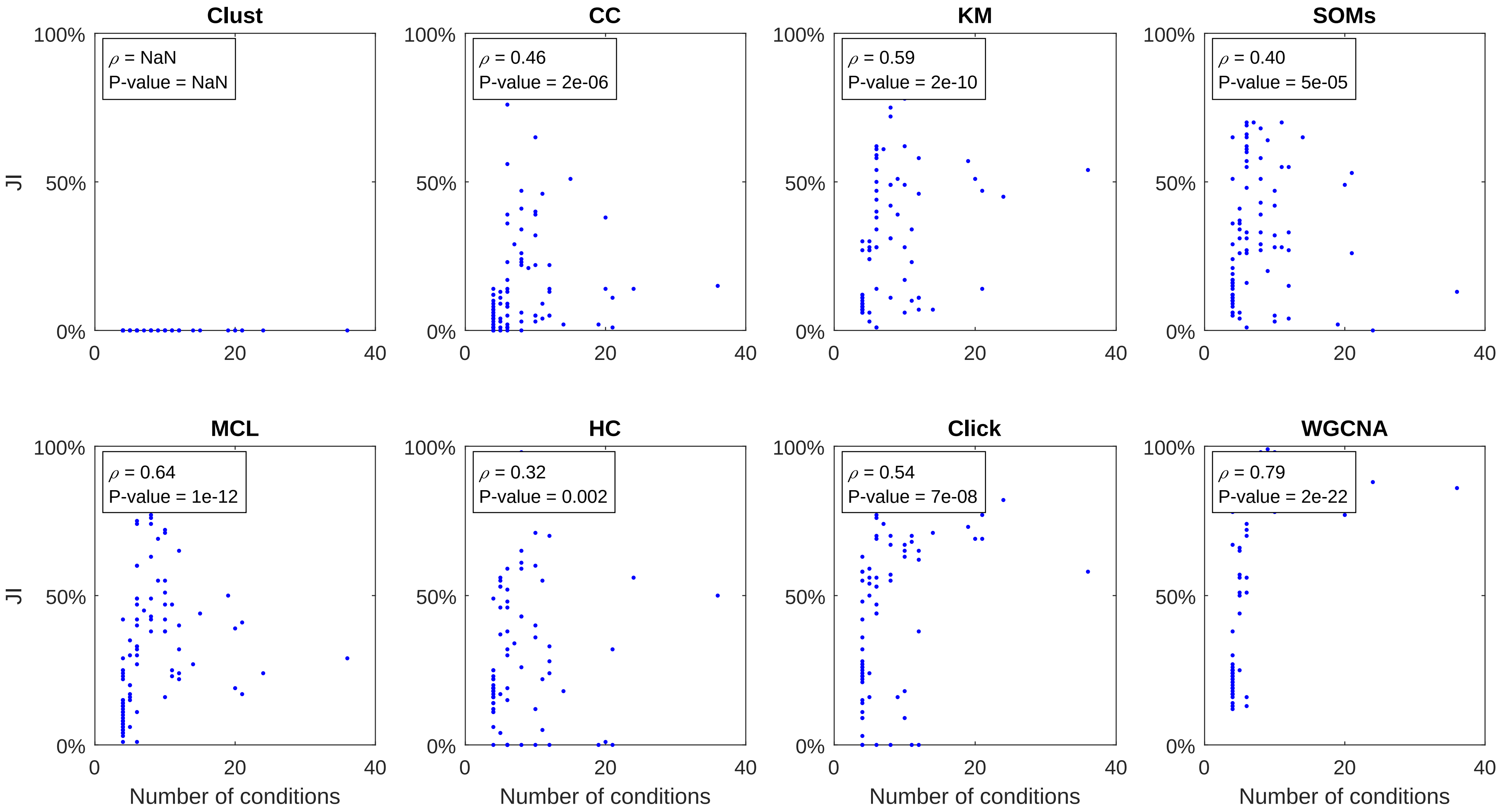

Figure S11 - JI versus number of conditions (samples) in the dataset analysis

Each scatter plot shows 100 points corresponding to the clustering results of applying the named method to each one of the 100 datasets. That is, each point represents one whole clustering result. Each plot shows average percentage of cluster overlap (POG), measured by JI, on the vertical axis, and the number of conditions (samples) in the dataset on the horizontal axis. Spearman's correlation values (ρ) between JI and the number of conditions are shown alongside their associated p-values.

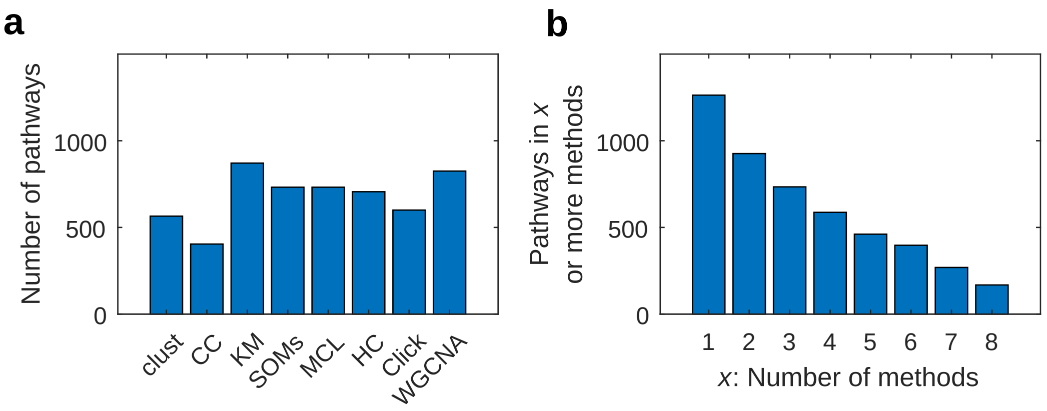

Figure S12 - Analysis of REACTOME pathway enrichment

(a) The total numbers (sum) of REACTOME pathways detected as significantly enriched in the results of each of the eight methods across the 20 selected datasets. (b) Numbers of pathways detected as significantly enriched in the same dataset by *x* or more methods; over the 20 datasets, 1,263 pathways were detected by at least one method, 337 (27%) of which are exclusive to a single method, and only 168 (13%) pathways were unanimously agree by all eight methods.

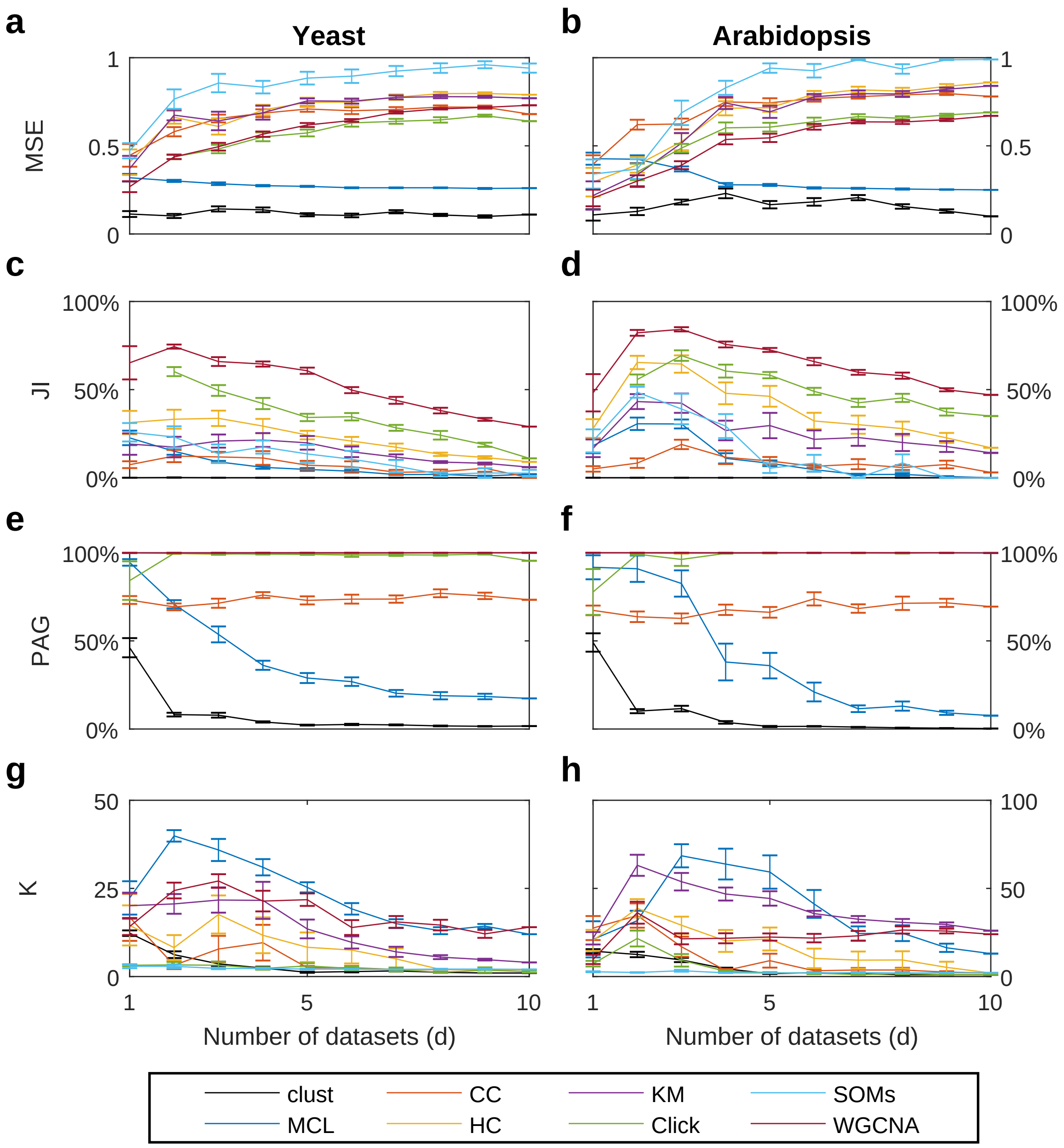

Figure S13 - Analysis of clustering multiple datasets simultaneously

Each sub-plot shows one performance metric measured for each method when applied on (*d*) different datasets simultaneously. Horizontal axes represent the numbers of datasets clustered clustered (*d*), vertical axes represent the performance metrics' values, the error bars represent standard error values over 10 random repetitions. The datasets are from either yeast (a, c, e, and g) or arabidopsis (b, d, f, and h). The performance metrics are within-cluster dispersion measured by MSE (smaller values are better) (a & b), percentage of overlap amongst clusters measured by the JI index (smaller values are better) (c & d), percentage of genes assigned to clusters (PAG) (e & f), and number of clusters generated (K) (g & h).
