# Supplementary material for: Clust: automatic extraction of optimal co-expressed gene clusters from gene expression data
Source: Genome Biol. 2018 Oct 25;19:172. doi: 10.1186/s13059-018-1536-8 (PMC6203272; doi:10.1186/s13059-018-1536-8)
Supplement: Supplementary file 3 — Text S1. The full description of the clust algorithm. (PDF 353 kb) [file 13059_2018_1536_MOESM3_ESM.pdf]

1 The *Clust* Method

Clust is an automatic cluster extraction method that can be applied to a single gene expression dataset or multiple datasets to extract the clusters of co-expressed genes. If a multiple datasets are considered, a cluster of co-expressed genes is defined as a set of genes which are co-expressed with each other in all of those datasets. This document describes the technical details of *clust*.

1.1 *Clust* workflow overview

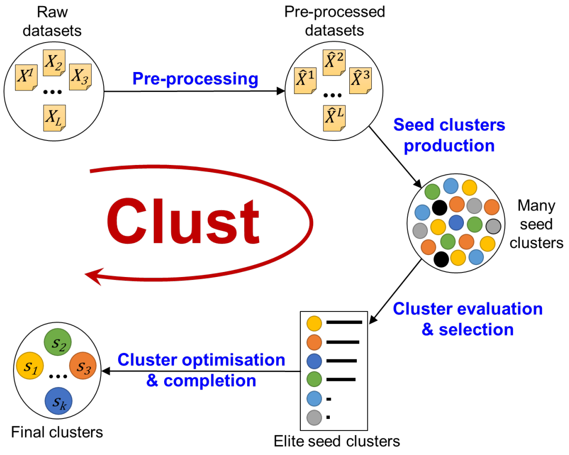

Figure 1: The automatic workflow of *clust*

Figure 1 shows the workflow of *clust*. After *clust* receives the raw dataset(s), it pre-processes them by filtering, summarisation, and normalisation. Then, a large number of seed clusters are produced by the Bi-CoPaM method (binarisation of consensus partition matrices). The M-N scatter plots technique is thereafter applied to select an elite subset of these seed clusters. Finally, a cluster optimisation and completion algorithm is employed to exploit the information in the elite seed clusters in order to produce the final largest, tightest, and non-overlapping clusters of co-expressed genes. Algorithm 1 shows the top-level *clust* procedure, and the next section defines the variables and symbols that are used throughout this document.

Algorithm 1 Clust

procedure CLUST($\mathbf{X}$, CM={KM}, K=4:2:20, Δ=0:0.1:1, t=1.0, sc=11, $Q3s$=2.0)
    $\mathbf{X}$ = Preprocess($\mathbf{X}$)
    R^{seed} = Bicopam($\mathbf{X}$, CM, K, Δ)
    (R^{elite}, MND) = MN($\mathbf{X}$, R^{seed}, t, sc)
    R^{final} = Optimise($\mathbf{X}$, R^{elite}, MND, sc, $Q3s$)
    Return R^{final}
end procedure

1.2 Definition of variables and symbols

This section lists the variables and symbols that are used throughout the description of the steps of *clust*. Few other symbols are used locally in some steps of *clust* and are defined where they are used.

Table 1: Definition of variables and symbols

Symbol	Description
$\mathbf{X}$	Set of datasets $\{X^l, l \in 1{:}L\}$
X^l	The lth dataset, which is a matrix of (Ng genes $\times$ D^l dimensions)
CM	List of base clustering methods; default: {k-means}. Other possible methods include SOMs and HC.
t	Tightness parameter; default: 1; tighter clusters: $t > 1$; wider clusters: $0 < t < 1$
sc	Size of the smallest allowed cluster $\in \mathbb{N}^+$; default: 11
$Q3s$	Number of 3rd quartiles for the optimisation threshold $\in \mathbb{R}^+$; default: 2.0
l, d, g, s, k, δ, and c	Indices always used to refer to a dataset (l), a condition (d), a gene (g), a cluster (s), number of clusters in a set of clusters (k), a binarisation parameter (δ) value, and a clustering method (c), respectively
L	Number of datasets
D^l	Number of conditions (dimensions) in the lth dataset
Ng	Total number of genes in the dataset(s)
K	Set of k values $\{k^1 \; ... \; k^{N_k}\}$ for base clustering methods (default: {4, 6, 8, ... , 20})
N_k	Number of k values
N_c	Number of base clustering methods (e.g. k-means)
Δ	Set of δ values
$\mathbf{x}_g^l$	A vector of the gene expression values of the gth gene in the lth dataset across its D^l conditions
$x_{g,d}^l$	The gene expression of gth gene at the dth condition of the lth dataset
R	A set of clusters (e.g. clustering result). This is a matrix in which columns represent clusters (Ng genes $\times$ k clusters)
$r_{g,s}$	An element of a set of of clusters (R) which represents the membership of the gth gene in the sth cluster. If this clustering result is fuzzy, $r_{g,s} \in [0, 1]$, and if it is binary, $r_{g,s} \in 0, 1$
R^{seed}, R^{elite}, and R^{final}	Sets of seed clusters, elite clusters, and final clusters, respectively. Each has the structure of R
MND	List of the M-N distances of the clusters in R^{elite}
MND_s	M-N distance of the sth cluster $\in [0, \sqrt{1 + t^2}]$

1.3 Seed cluster production (The Bi-CoPaM method)

The Bi-CoPaM (binarisation of consensus partition matrices) method (Algorithm 2) is used to produce a large pool of seed clusters. Given a fixed number of clusters (k value), a set of datasets ($\mathbf{X}$), a set of base clustering methods (CM), Bi-CoPaM applies each one of the base clustering methods to each one of the datasets to produce multiple partitions (clustering results). Then, a consensus fuzzy consensus clustering result (FCR) is calculated from these different clustering results. In FCR, each gene is assigned to each one of the k clusters with a fuzzy membership value $\in [0, 1]$, where 1.0 means it fully belongs to that cluster, 0.0 means it does not belong to it at all, and a value between 0.0 and 1.0 means it partially belongs. Finally, FCR is binarised by the difference threshold binarisation (DTB) algorithm to produce a binary consensus result (BCR) in which membership values are strictly either 1.0 (belongs) or 0.0 (does not belong). Note that the DTB binarisation algorithm requires setting a tuning parameter $\delta \in [0.0, 1.0]$.

Thus, given a fixed k value and a fixed δ value, the Bi-CoPaM produces k consensus clusters, which we call *seed clusters*. In order to explore the space of k and δ values, the Bi-CoPaM produces different versions of seed clusters at every parameter pair (k, δ) taken from a given set of k values (K) and a given set of δ values (Δ). By default, $K = \{4, 6, 8, ..., 20\}$ and $\Delta = \{0.0, 0.1, ..., 1.0\}$. The pool of seed clusters eventually produced by Bi-CoPaM (R^{seed}) therefore includes all of the individual seed clusters produced at all of these (k, δ) parameter pairs.

Algorithm 2 Bi-CoPaM: seed cluster production

procedure BICOPAM($\mathbf{X}$, CM={KM}, K=4:2:20, Δ=0:0.1:1)
  $R^{seed} = ()$
  for each k value in K **do**
    for each dataset X^l in $\mathbf{X}$ **do**
      for each clustering method CM^c in CM **do**
        $R_k^{l,c} = \text{Apply}(CM^c, X^l, k)$
      end for
    end for
    $FCR_k = \text{FindConsensus}(\mathbf{R}_k)$
    for each δ in Δ **do**
      $BCR_k^\delta = \text{Binarise}(FCR_k, \delta)$
      $R^{seed} = \text{ConcatenateColumns}(R^{seed}, BCR_k^\delta)$
    end for
  end for
  Return R^{seed}
end procedure

▷ $R_k^{l,c}$: ($Ng \times k$) matrix; result of applying the cth method to the lth dataset at k
▷ $\mathbf{R}_k \equiv \{R_k^{l,c} \mid \forall l \forall c\}$: $L \times C$ different clustering results at the given k value
▷ FCR_k: fuzzy consensus result at k; ($Ng \times k$) matrix
▷ $FCR_k[g, s] \in [0, 1]$: fuzzy membership of the gth gene in the sth cluster
▷ BCR_k^δ: binary consensus result at k and δ; ($Ng \times k$) matrix
▷ $BCR_k^\delta[g, s] \in \{0, 1\}$: binary membership of the gth gene in the sth cluster
▷ R^{seed}: horizontal concatenation of BCR_k^δ matrices $\forall k \in K$, $\forall \delta \in \Delta$

1.3.1 Calculating the consensus fuzzy result *FCR*

Algorithm 3 is the algorithm which is used by the Bi-CoPaM to calculate the fuzzy consensus result ((FCR)) from a set of base clustering results $\mathbf{R}$. Calculating *FCR* from a set of clustering results $\mathbf{R} = \{R^1 ... R^{N_r}\}$ is done by straightforward averaging of these results. For example, if a gene is assigned to a cluster in 70% of the base clustering results and to another cluster in 30% of the results, it will have the membership value of 0.7 in the former cluster and 0.3 in the latter cluster. However, as per the nature of clustering algorithms, if there are two clustering results for the same dataset, it is not guarranteed that the sth cluster from in the first result corresponds to the sth cluster in the second result. Therefore, the k clusters in each one of these clustering results must be relabelled (re-ordered) so that the sth cluster in any of the results R^r corresponds to the sth cluster in each one of the other results $\{R^1 ... R^{N_r}\}$.

Relabelling (reordering) the clusters in a clustering result R to correspond to the clusters in a reference clustering result R^{ref} is done by following a min-min approach. Pairwise distances are calculated between every pair of clusters in those two clustering results. Then, the two clusters in the best matching pair (the one with the minimum distance) are mapped to each other. After that, these already-mapped clusters are eliminated from the matrix of pairwise distances allowing for the same step to be applied in the following iteration in order to select the second best matching pair. This iterative approach runs until all k clusters in R are mapped/aligned to the k clusters in R^{ref}.

In practice, as shown in Algorithm 3, *FCR* is initially assigned the values of the first clustering result R^1. Then, the second clustering result R^2 is relabelled against this initial version of *FCR*, and is merged with it by an element-by-element averaging to form an updated *FCR*. This process iterates over all of the clustering results, one-by-one, to relabel each of them against the current version of *FCR* and to update *FCR* accordingly. In fact, the order in which these clustering results are taken affects the result of this algorithm, where those taken first influence the final result more. Therefore, the N_r clustering results $\{R^1 ... R^{N_r}\}$ are first reordered in an ascending order based on the average mean-squared error (MSE) values of their clusters. MSE values, as explained in the following section, evaluate the dispersion within clusters, and thus lower MSE values reflect tighter (better) clusters. This reordering of the clustering results guarantees that better results are merged earlier in *FCR* and are therefore more influential over the final result.

Algorithm 3 Find the consensus of a set of partitions

```
procedure FindConsensus(R)
    ReorderMSE(R)
    FCR = R^1
    for r from 2 to N_r do
        R^{r*} = Relabel(FCR, R^r)
        FCR = ((r − 1) × FCR + R^{r*}) / r
    end for
    Return FCR
end procedure
                    ▷ R: set of clustering results with the same number of clusters (k)
                    ▷ R: {R^r | r ∈1:N_r; N_r: number of clustering results in R}
                    ▷ R^r: r^th clustering result; (Ng genes × k clusters) matrix
                    ▷ R^r[g, s] ∈ [0, 1]: membership of the g^th gene in the s^th cluster in the r^th clustering result
                    ▷ R^{r*}: relabelled version of R^r; (Ng genes × k clusters) matrix
                    ▷ ReorderMSE: reorder {R^1...R^{N_r}} in an ascending way based on their mean MSE values

procedure Relabel(R^ref, R)
    D = pdist(R^ref, R)
    max_distance = max(D)
    R* = emptymatrix(Ng×k)
    for i from 1 to k do
        (m,n) = argmin(D)        ▷ m & n are the row & the column of the minimum value in D
        R*[n^th column] = R[m^th column]
        D[m^th row] = max_distance
        D[n^th column] = max_distance
    end for
    Return R*
end procedure
                    ▷ R^ref: reference clustering result; (Ng genes × k clusters) matrix
                    ▷ R: clustering result to be relabelled based on R^ref; (Ng genes × k clusters) matrix
                    ▷ R*: relabelled version of R; (Ng genes × k clusters) matrix
                    ▷ pdist: function to find the pairwise distance between the columns of two matrices
                    ▷ D: (k×k) matrix of pairwise distances between the k clusters in R^ref and R
```

1.3.2 The DTB binarisation algorithm

Algorithm 4 describes the DTB binarisation algorithm. This algorithm transforms every fuzzy membership value ($\in [0.0, 1.0]$) in the fuzzy consensus result matrix (FCR) into a binary membership value (either fully belongs (1) or does not belong at all (0)). The algorithm performs this in a gene-by-gene manner, where each gene is represented by a row in the FCR matrix. Given the fuzzy membership values of a given gene in all of the clusters, it is assigned to the cluster in which it has its highest fuzzy membership only if it is larger than its second highest membership with a difference of δ or more. On the other hand, if the highest two membership values of that gene

are closer to each other than δ, it is assumed that there is no sufficient evidence for this gene to be exclusively included in a particular cluster, and is therefore not assigned to any of the clusters. The parameter $\delta \in [0.0, 1.0]$ is a tuning parameter which controls the strictness of binarisation. The tightest (most strict) results are obtained at $\delta = 1.0$ where a gene is assigned to a cluster only if its fuzzy membership in it is 1.0 and is 0.0 in all other clusters. The least strict results are obtained at $\delta = 0.0$ where every gene is assigned to the cluster in which it has its highest membership value regardless of its other membership values. In the latter case, ties may happen between clusters competing over a gene, the case in which a gene is assigned to all of these equally competing clusters in the binarised result.

Algorithm 4 Difference threshold binarisation (DTB) algorithm

```
procedure BINARISE(FCR, δ)
    BCR = zerosmatrix(Ng×k)                                  ▷ Initialise BCR with zeros
    for g from 1 to Ng do
        top_cluster_index = argmax(FCR[g^th row])
        top_cluster_membership = max(FCR[g^th row])
        second_top_cluster_membership = secondmax(FCR[g^th row])
        if (top_cluster_membership - second_top_cluster_membership) ≥ δ) then
            BCR[g, top_cluster_index] = 1
        else
            Skip to next gene                                ▷ Gene is not assigned to any cluster
        end if
    end for
    Return BCR
end procedure
                          ▷ FCR: fuzzy consensus result; (Ng genes × k clusters) matrix
                          ▷ BCR: binary consensus result; (Ng genes × k clusters) matrix
```

1.4 Elite seed cluster selection (The M-N scatter plots technique)

Algorithm 5 describes the M-N scatter plots technique which selects an elite set of non-overlapping clusters out of the large set of seed clusters generated by the Bi-CoPaM method under varying parameter values. Each seed cluster (s) is evaluated using two metrics:

1. $M_s^* \in [0.0, t]$: measures the **dispersion** as a scaled value of the mean-squared error (MSE) of the genes in that cluster. In the case of multiple datasets, the MSE is first calculated for the cluster s based on each one of the L datasets ($M_s^1 ... M_s^L$). Then, the maximum of these values is considered as the representative MSE of the cluster (M_s). As larger MSE values indicate higher dispersion, and therefore less tight clusters, considering the maximum in this case means that the quality of the cluster here is limited by its worst performance across the datasets. The final M_s^* values are calculated for all clusters after that by linearly transforming all M_s values to the range $[0.0, t]$. **Better clusters have smaller M_s^* values**.

2. $N_s^* \in [0.0, 1.0]$: measures the **size** of the cluster. This is the number of genes in the cluster after being transformed to the logarithmic scale first and then to the $[0.0, 1.0]$ range. **Better clusters have larger N_s^* values**.

Better clusters are those with smaller dispersion while including more genes, and therefore are those which minimise M_s^* and maximise N_s^*. If all clusters are scattered as points on a 2D plot whose horizontal axis is M_s^* and whose vertical axis is N_s^*, better clusters will be those closer to the top left corner of the plot with the coordinates $(0.0, 1.0)$. So, the single-value metric which reflects the quality of a cluster s according to this algorithm is its distance from the top left corner of the M-N plot. This distance is called the M-N distance (MND_s), and better clusters have smaller MND_s values.

Clearly, the M-N scatter plot has the shape of a $(1.0\times t)$ rectangle. Indeed, varying the parameter t changes the weight with which the horizontal dimension M_s^* affects the result compared with the vertical dimension N_s^*. Higher t values favour tighter (less dispersed) clusters while compensating the size of the cluster while smaller t values have an opposite effect. The default value of t is 1.0.

After finding the MND_s value for all clusters, the cluster with the smallest MND_s value is selected as the first and best elite seed cluster. After that, all of the seed clusters which have genes shared with that selected seed cluster are eliminated. The same steps are repeated over the remaining seed clusters. That is, to select the cluster with the minimum MND_s and then to eliminate the clusters that share genes with it. The process is repeated until no seed clusters remain. Each of these iterations identifies a single elite seed cluster. It can be clearly seen that this iterative process produces a list of elite seed clusters in order of quality. Finally, if any selected clusters are smaller than the pre-specified smallest cluster size (sc), they are eliminated.

Algorithm 5 M-N plots selection

procedure MN($\mathbf{X}$, R^{seed}, t=1.0, sc=11)
  for each cluster s in R^{seed} **do**
    for each dataset X^l in $\mathbf{X}$ **do**
      $M_s^l = \frac{1}{D_l \times N_s} \sum_{\mathbf{x}_g^l, \forall g \in C_s} ||\mathbf{x}_g^l - \mathbf{z}_s^l||^2$
    end for
    $M_s = \max_{\forall l} \left(M_s^l \right)$
  end for
  for each cluster s in R^{seed} **do**
    $M_s^* = t \times \left(\frac{M_s - \min_{\forall s} \left(M_s \right)}{\max_{\forall s} \left(M_s \right) - \min_{\forall s} \left(M_s \right)} \right)$
    $N_s^* = \frac{\log \left(N_s \right)}{\max \left(\log \left(N_s \right) \right)}$
  end for
  $MND_s = \sqrt{{M_s^*}^2 + \left(1 - N_s^*\right)^2}$
  $EliteClusters = ()$
  $RemainingSeedClusters = 1{:}\hat{k}$
  i=1
  while $RemainingSeedClusters$ is not empty **do**
    $bestcluster = \arg\min_{\forall s \in RemainingSeedClusters} \left(MND_s \right)$
    $EliteClusters[i] = bestcluster$
    Remove($RemainingSeedClusters$, $bestcluster$)
    $SimilarClusters = \{ s | (cluster_s \cap cluster_{bestcluster}) \neq \emptyset \}$
    Remove($RemainingSeedClusters$, $SimilarClusters$)
    $i = i + 1$
  end while
  $R^{elite} = R^{seed}$[columns: $EliteClusters$]
  RemoveSmallClusters(R^{elite}, sc) ▷ Remove clusters with less than sc genes
  Return (R^{elite}, MND)
end procedure

▷ M_s^l: MSE value of the sth cluster based on the lth dataset
▷ M_s: Summarised MSE value of the sth cluster based on all datasets
▷ $M_s^* \in [0, t]$: Scaled MSE value of the sth
▷ N_s: number of genes in the sth cluster
▷ $N_s^* \in [0, 1]$: scaled log number of genes in the sth cluster
▷ $C_s \subset (1 : N_g)$: set of indices of the genes in the sth cluster
▷ $\mathbf{x}_g^l$: vector of gene expression values of the gth gene in the lth dataset
▷ $\mathbf{z}_s^l$: average gene expression values of the genes in the sth cluster in the lth dataset

1.5 Cluster optimisation and completion

This step of the *clust* aims at addressing some of the defects that can be found in the selected elite seed clusters (Algorithm 6). In particular, three issues are addressed by this algorithm:

1. False positives: These are genes that are included in clusters but they do not fit well within their profiles. For instance, outliers are among false-positives.
2. False negatives: These are genes which fit well within some cluster's profile but are not included in it.
3. Profiles' overlap: These are genes which fit well within the profiles of two or more clusters at the same time. In reality, each of these genes might be included in one or none of these clusters, but it fits within the other cluster(s).

Addressing these issues collectively is done by following this rationale:

1. The elite seed clusters are ordered based on their *MND* values. So split them into "good clusters" and the "rest of the clusters" by the "largest-weighted-gap" technique (described below).
2. From the "good clusters", empirically learn the accepted dispersion of gene expression values from their mean by using the "modified one-tailed Tukey's method" (described below).
3. Go back to all elite seed clusters and consider their average profiles as skeletons for the final optimised clusters.
4. Temporarily define the boundaries of the final clusters as their average profiles $\pm$ the maximum accepted dispersion. These average profiles and accepted dispersion values have been empirically learnt in the steps above.
5. Update the boundaries of the final clusters by resolving any overlaps between any two defined cluster-boundaries by the algorithm described below.
6. Ignore the original assignment of genes to clusters, and freshly map all genes in the data that fully fit within any of the defined clusters' boundaries to them. This defines the final cluster membership.

As can be seen, this optimisation algorithm considers all of the previous steps that produced elite seed clusters as an exercise to empirically learn the shapes of the gene expression profiles that exist in the data as well as the acceptable dispersion around these shapes. Once such empirically derived cluster-boundaries are identified, genes are assigned to them.

It can also be seen that this approach takes care of the three aforementioned issues. False positives (outliers) will not be included in the final clusters because they will not fit within the boundaries that were learnt empirically. Importantly, the technique used to learn the maximum acceptable dispersion, and therefore the boundaries, is robust to outliers (see the modified one-tailed Tukey's method described below). As for false-negatives, the algorithm includes all genes

that fit well within the boundaries of the clusters in them by a brute force iteration over all genes. This guarantees that no gene which fits well within a cluster, compared to the other genes in that cluster, will be missed. Finally, the boundaries are guaranteed not to overlap by the overlap-resolving method described below, and this addresses the third issue.

Algorithm 6 Cluster optimisation and completion

procedure OPTIMISE($\mathbf{X}$, R^{elite}, MND, sc=11, $Q3s$=2.0)
  $GoodClusters$ = LargestWeightedGap(MND)
  for each dataset X^l in $\mathbf{X}$ **do**
    for each gene g in 1:Ng **do**
      s = the cluster to which the gene g belongs
      if ($s \in GoodClusters$) **and** ($\mathbf{z}_s^l$ is not an all-zeros vector) **then**
        $\mathbf{e}_g^l = |\mathbf{x}_g^l - \mathbf{z}_s^l|$, where gene $g \in s^{th}$ cluster
      else
        Remove $\mathbf{e}_g^l$ from E^l
      end if
    end for
    $\mathbf{q3}^l$ = Columnwise3rdQuartile(E^l)
    $\mathbf{emax}^l = Q3s \times \mathbf{q3}^l$
    for each cluster s in 1:k^{elite} **do**
      $\mathbf{lower}_s^l = \mathbf{z}_s^l$ - $\mathbf{emax}^l$
      $\mathbf{upper}_s^l = \mathbf{z}_s^l + \mathbf{emax}^l$
    end for
  end for
  (**Lower**, **Upper**) = ResolveOverlaps(**Lower**, **Upper**)
  $R^{final} \equiv (Ng \times k^{elite})$ matrix; $r_{g,s}^{final} = \begin{cases} 1, & lower_{s,d}^l \le x_{g,d}^l \le upper_{s,d}^l \ \forall l \ \forall d \\ 0, & \text{otherwise} \end{cases}$
  RemoveSmallClusters(R^{final}, sc) ▷ Remove clusters with less than sc genes
  Return R^{final}
end procedure

▷ R^{elite} are in an ascending order based on their M-N distances (MND)
▷ $GoodClusters$: a list of indices of the "good clusters"

1.5.1 Largest-weighted-gap technique

The elite seed clusters are ordered based on their MND values. So, the ordered MND values are used to split the clusters into "good clusters" and the "rest of the clusters". The largest-weighted-gap technique calculates the gaps (the differences) between every two consecutive clusters' MND values. The gaps are then weighted such that the last gap, which is the one between the last and the next-to-last clusters, has zero weight, and the weights increase with natural numbers for earlier gaps. Then, the clusters before the largest weighted gap are defined as the "good clusters".

Algorithm 7 Finding "good clusters": Largest-weighted-gap technique

```
1: procedure LargestWeightedGap(MND)
2:     gaps = MND_{2:k^elite} - MND_{1:(k^elite - 1)}
3:     wgaps = gaps × ((k^elite − 1) : 1)
4:     GoodClusters = 1:argmax(wgaps)
5:     Return GoodClusters
6: end procedure
```

▷ k^{elite}: number of elite clusters; this is equal to the length of *MND*

▷ $MND_{a:b}$: a subset of *MND* from the ath element to the bth, inclusively

1.5.2 Modified one-tailed Tukey's method

This is a method to identify the maximum accepted dispersion of gene expression values from their average empirically based on the genes in the "good clusters". This maximum dispersion is defined independently for each dimension (condition) in each dataset, and is calculated as the absolute difference from the mean at that dimension. Given the dth condition in the lth dataset, dispersion values e are calculated for all of the genes in all of the "good clusters". The 3rd quartile of these e values is identified after that ($q3$), and the maximum accepted dispersion is calculated as $emax = Q3s \times q3$, where $Q3s$ is a used-defined parameter with the default value of 2.0.

The parameter $Q3s > 0$ defines how many 3rd quartiles far from the mean are accepted. Thus, higher values of $Q3s$ allow for less tight clusters to be formed, while smaller values force clusters to be tighter. All values beyond $Q3s \times q3$ are considered as outliers.

Having calculated the maximum allowed dispersion values $emax$ for each dimension (d) in each dataset (l), the upper and the lower boundaries of the clusters are defined as their average profiles $\pm$ these $emax$ values. Based on that, a gene is said to fit within a cluster's profile if its expression values are between the upper and the lower boundaries of that cluster at every condition of every dataset. If it fails to fulfill this criterion even at a single condition, the gene is not said to fit within this cluster.

1.5.3 Resolve overlaps among clusters' boundaries

The clusters' upper and lower boundaries are investigated for any overlaps, which are then eliminated as shown in Algorithm 8. An overlap between two clusters exists if their boundaries are defined in way that makes it possible for some gene expression profile to fully fit within both of them. This only applies if the overlap appears across all of the conditions in all of the datasets.

The problem is resolved by eliminating the overlap between the two clusters at a single condition by redefining the boundaries of one of the two clusters therein. The condition (dimension) to be fixed in this case is the one with the smallest overlap because it will result in minimum interference. Moreover, as the clusters are ordered based on their original *MND* values, the cluster to be modified in this case is the cluster which is latter in order. This makes such interference has a smaller effect over better clusters. Finally, the boundary update is simply done by redefining the overlapping boundary at the chosen condition of the chosen cluster to be just outside the boundaries of the other cluster at that condition.

Algorithm 8 Resolve overlaps between clusters' boundaries

```
procedure ResolveOverlaps(Lower, Upper)
    for s1 in 1:k^{elite} do
        for s2 in 1:k^{elite} do
            if s1 ≥ s2 then Skip to next iteration end if
            v_ov = 0                         ▷ Value of the smallest overlap
            l_ov = -1                        ▷ Dataset of the smallest overlap
            d_ov = -1                        ▷ Dimension of the smallest overlap
            t_ov = -1                        ▷ Type of the smallest overlap ∈ {-1, 0, 1, 2}
            for l in 1:L do
                for d in 1:D_l do
                    if (upper^l_{s1,d} > lower^l_{s2,d} and upper^l_{s1,d} ≤ upper^l_{s2,d}) then
                        if lower^l_{s1,d} < lower^l_{s2,d} then
                            ov = upper^l_{s1,d} - lower^l_{s2,d}
                            if (t_ov == -1 or ov < v_ov) then
                                t_ov = 0
                                v_ov = ov
                                l_ov = l
                                d_ov = d
                            end if
                        end if
                    else if (upper^l_{s2,d} > lower^l_{s1,d} and upper^l_{s2,d} ≤ upper^l_{s1,d}) then
                        if lower^l_{s2,d} < lower^l_{s1,d} then
                            ov = upper^l_{s2,d} - lower^l_{s1,d}
                            if (t_ov == -1 or ov < v_ov) then
                                t_ov = 1
                                v_ov = ov
                                l_ov = l
                                d_ov = d
                            end if
                        end if
                    else   t_ov = 2; Skip to next iteration
                    end if
                end for
                if (t_ov == 2) then Skip to next iteration end if
            end for
            if (t_ov == −1) then    lower^0_{s2,0} = 1;    upper^0_{s2,0} = 0
            else if (t_ov == 0) then    lower^{l_ov}_{s2,d_ov} = upper^{l_ov}_{s1,d_ov} + ϵ
            else if (t_ov == 1) then    upper^{l_ov}_{s2,d_ov} = lower^{l_ov}_{s1,d_ov} − ϵ
            end if
        end for
    end for
    Return (Lower, Upper)
end procedure
```
